# Topical Vitamin D Analogs Beyond Psoriasis: Pharmacological Mechanisms, Cutaneous Drug Delivery, and Clinical Evidence

**DOI:** 10.3390/pharmaceutics18101248

**Published:** 2026-10-01

**Authors:** Doris Laçej, Anna Pietrella, Nicole Zoratto, Marianna Lombardi, Chiara Di Meo, Pietro Matricardi

**Affiliations:** 1 Department of Drug Chemistry and Technologies, Sapienza University of Rome, Piazzale Aldo Moro, 5, 00185 Rome, Italy; doris.lacej@uniroma1.it (D.L.); marianna.lombardi@uniroma1.it (M.L.); chiara.dimeo@uniroma1.it (C.D.M.); 2 Department of Biology, Tor Vergata University of Rome, Via della Ricerca Scientifica 1, 00133 Rome, Italy; anna.pietrella@uniroma2.it (A.P.); nicole.zoratto@uniroma2.it (N.Z.)

**Keywords:** vitamin D analogs, topical drug delivery, cutaneous delivery, topical formulations, calcipotriol, tacalcitol, calcitriol, skin targeting

## Abstract

Topical vitamin D analogs are well-established therapies for psoriasis, but their broader dermatologic potential increasingly depends not only on their biological activity but also on the ability of topical formulations to achieve effective and localized cutaneous delivery. This narrative review critically examines the emerging use of calcipotriol, tacalcitol, and calcitriol beyond psoriasis, with particular emphasis on formulation strategies, cutaneous drug delivery, and their relationship with clinical performance. Conventional topical dosage forms, including ointments, creams, and solutions, remain the principal delivery systems used in clinical studies. Their therapeutic performance is influenced by vehicle composition, skin-barrier integrity, local retention, application conditions, and the physicochemical properties of the vitamin D compound, which collectively determine cutaneous penetration, tolerability, and systemic exposure. Across the reviewed indications, formulations and treatment protocols vary considerably, contributing to heterogeneity in clinical outcomes. Vitiligo currently presents the strongest clinical evidence, particularly when topical vitamin D analogs are combined with narrowband ultraviolet B phototherapy, whereas encouraging results have been reported for morphea, acne vulgaris, and inherited ichthyoses. Evidence for atopic dermatitis, alopecia areata, and basal cell carcinoma remains limited or inconsistent. Beyond conventional vehicles, emerging delivery technologies including liposomes, nanoemulsions, solid lipid nanoparticles, nanostructured lipid carriers, polymeric nanocarriers, hydrogels, and microneedle-assisted systems offer opportunities to enhance skin targeting, improve local drug retention and reduce systemic exposure. However, evidence specifically evaluating these advanced systems for vitamin D analogs remains predominantly preclinical. Future research should integrate clinical evaluation with formulation optimization, cutaneous pharmacokinetics, and comparative delivery studies, as accumulating evidence suggests that formulation-dependent skin delivery may be a critical determinant of therapeutic success beyond psoriasis.

## 1. Introduction

Vitamin D is increasingly recognized as a multifunctional signaling molecule whose biological activity extends far beyond its classical role in calcium and phosphate homeostasis. The discovery that the vitamin D receptor (VDR) is widely expressed in both immune and non-immune tissues has transformed our understanding of vitamin D from a regulator of mineral metabolism into a pleiotropic mediator of cellular proliferation, differentiation, apoptosis, and immune homeostasis. Among human organs, the skin occupies a unique position because it functions simultaneously as the principal site of endogenous vitamin D synthesis and as a major target of vitamin D signaling. Following ultraviolet B (UVB) exposure, epidermal 7-dehydrocholesterol is converted to vitamin D_3_, which subsequently undergoes sequential hydroxylation to form the biologically active metabolite, 1,25-dihydroxyvitamin D_3_ (calcitriol). In addition to this classical endocrine pathway, keratinocytes possess the enzymatic machinery required for local activation and degradation of vitamin D, establishing an autonomous cutaneous vitamin D system capable of regulating epidermal homeostasis independently of circulating vitamin D concentrations. Ligand binding to the VDR induces heterodimerization with the retinoid X receptor (RXR) and subsequent interaction with vitamin D response elements (VDREs), regulating genes involved in epidermal differentiation, immune homeostasis, oxidative stress responses, tissue remodeling, and cell survival. Consequently, VDR signaling influences virtually every major cutaneous cell population, including keratinocytes, melanocytes, sebocytes, dermal fibroblasts, and resident immune cells, providing a strong biological rationale for therapeutic intervention across a broad spectrum of inflammatory, pigmentary, hyperproliferative, fibrotic, and neoplastic skin diseases [1,2,3,4,5]. The recognition that vitamin D signaling regulates epidermal proliferation and differentiation prompted the development of synthetic vitamin D analogs specifically designed for topical dermatologic use. Although calcitriol, the naturally active form of vitamin D_3_, demonstrated potent biological activity in early experimental studies, its therapeutic application was limited by the risk of systemic hypercalcemia when administered at clinically relevant doses. Consequently, considerable efforts were directed toward developing synthetic analogs capable of preserving VDR-mediated biological activity while minimizing calcemic effects and improving pharmacokinetic properties suitable for long-term topical administration. These developments led to the clinical use of three principal topical vitamin D compounds in dermatology: the synthetic analogs calcipotriol (calcipotriene) and tacalcitol, together with calcitriol, the naturally active form of vitamin D_3_. Importantly, the therapeutic performance of topical vitamin D analogs is determined not only by their intrinsic pharmacological activity but also by the formulation and delivery system through which they are administered. Conventional formulations used clinically or investigated in dermatologic studies include ointments, creams, gels, lotions, solutions, and foams, each of which differs in drug release, occlusivity, skin hydration, cosmetic acceptability, and capacity to promote penetration across the stratum corneum. Ointment-based formulations may enhance local drug retention and penetration through their occlusive properties, whereas creams, gels, lotions, solutions, and foams may offer improved spreadability and patient acceptability, particularly on the face, scalp, or hair-bearing areas. Beyond conventional vehicles, advanced delivery platforms, including liposomes, nanoemulsions, solid lipid nanoparticles (SLNs), nanostructured lipid carriers (NLCs), polymeric nanoparticles, and hydrogels, are increasingly being investigated to improve cutaneous targeting, drug stability, controlled release, and local bioavailability while minimizing systemic exposure [6,7,8,9]. These formulation considerations are particularly relevant to the repurposing of vitamin D analogs because the desired site of drug delivery varies according to disease pathophysiology, ranging from the pilosebaceous unit in acne and the hair follicle in alopecia areata to the epidermal melanocyte compartment in vitiligo and deeper dermal tissue in morphea. Accordingly, formulation design and cutaneous drug delivery represent important determinants of therapeutic performance and should be considered alongside pharmacological activity when interpreting existing clinical evidence and developing future therapeutic strategies. Despite sharing a common mechanism of action through VDR activation, these molecules differ in chemical structure, receptor affinity, metabolic stability, local tolerability, and systemic calcemic potential. Such pharmacological differences may influence their suitability for specific anatomical sites, treatment duration, and disease indications. Regulatory indications vary according to compound, formulation, and jurisdiction. In the United Kingdom, currently authorized topical formulations of calcipotriol, calcitriol, and tacalcitol are indicated for psoriasis vulgaris/plaque psoriasis, with formulation-specific differences in disease severity, anatomical site, and treatment conditions [10,11,12]. The non-psoriatic dermatological applications discussed in this review are therefore considered off-label or investigational unless otherwise specified. Nevertheless, their pharmacological properties and broader biological effects have stimulated increasing interest in their potential therapeutic application beyond psoriasis.

**Figure 1:**
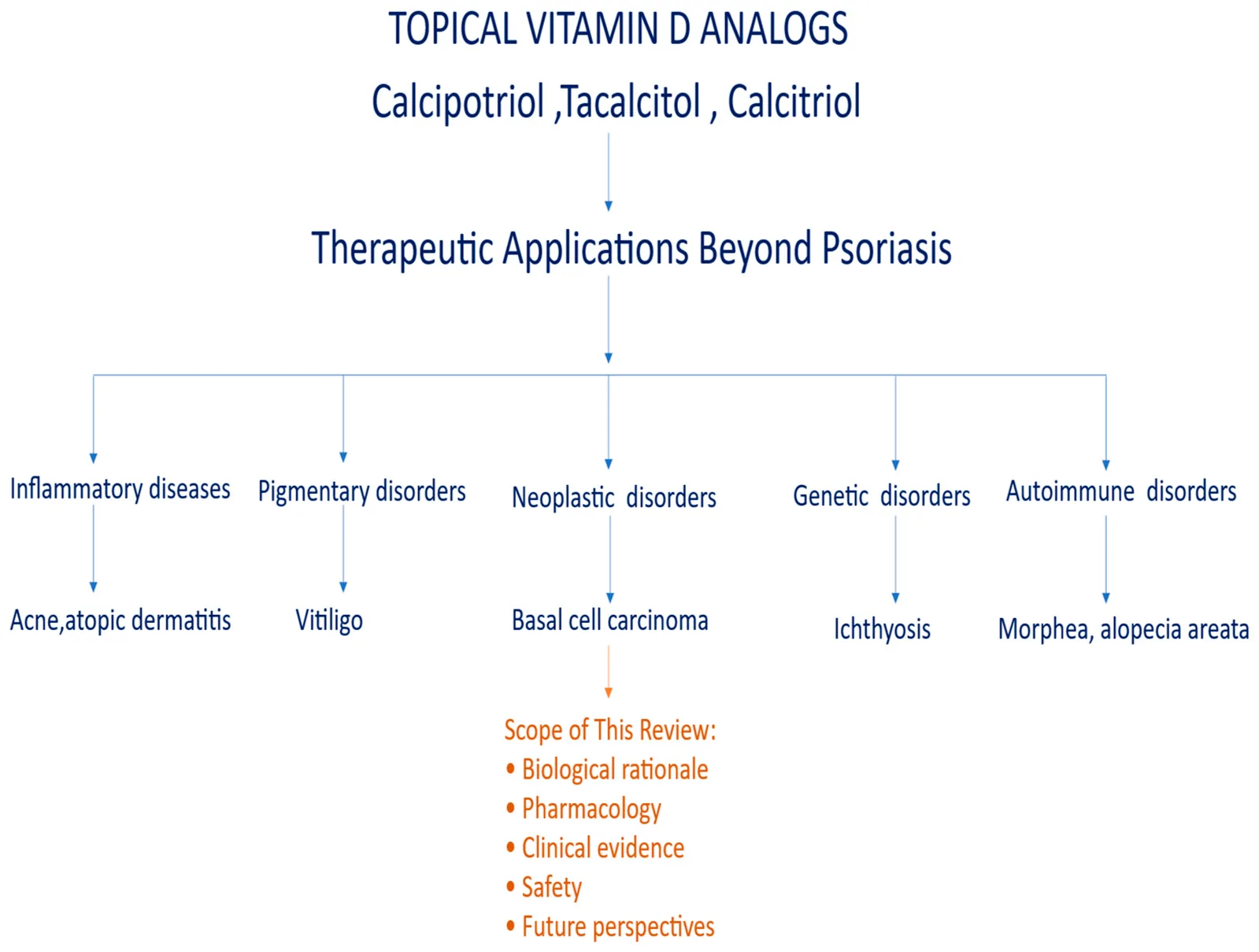
Spectrum of non-psoriatic dermatologic disorders.

Over the past two decades, advances in cutaneous immunology and molecular dermatology have substantially broadened our understanding of VDR biology. Beyond its well-established effects on keratinocyte proliferation, VDR signaling has been implicated in the regulation of innate and adaptive immune responses, epidermal barrier integrity, oxidative stress, melanocyte survival, sebocyte differentiation, extracellular matrix remodeling, and tumor cell biology. These diverse biological functions suggest that topical vitamin D analogs may offer therapeutic benefits across a spectrum of inflammatory, pigmentary, hyperproliferative, fibrosing, and neoplastic skin disorders that extend far beyond psoriasis. Accordingly, topical vitamin D analogs have been investigated in conditions including acne vulgaris, atopic dermatitis, vitiligo, basal cell carcinoma, inherited ichthyoses, morphea, alopecia areata, and several other less common dermatoses. The biological rationale varies according to disease pathogenesis, ranging from modulation of antimicrobial peptide expression and sebocyte activity in acne to regulation of melanocyte survival in vitiligo, suppression of transforming growth factor-β-driven fibrosis in morphea, and inhibition of oncogenic Hedgehog signaling in basal cell carcinoma. Collectively, these findings position topical vitamin D analogs as promising candidates for therapeutic repurposing in modern dermatology [1,2,13,14,15]. Despite increasing interest in the therapeutic potential of topical vitamin D analogs beyond psoriasis, the available evidence remains fragmented across diverse dermatologic conditions and study designs. Most published reviews have focused on individual diseases, systemic vitamin D supplementation, or the role of vitamin D deficiency in skin disorders, while comprehensive evaluations specifically addressing topical vitamin D analogs across multiple non-psoriatic indications remain lacking. Moreover, calcipotriol, tacalcitol, and calcitriol are frequently considered interchangeable despite important differences in their pharmacological characteristics, local tolerability, and potential clinical applications. Consequently, clinicians currently have limited evidence-based guidance regarding which analog may be most appropriate for specific diseases or anatomical sites. In this context, the present narrative review provides a comprehensive and critical synthesis of the available evidence regarding topical vitamin D analogs in non-psoriatic skin diseases (Figure 1). We summarize the mechanistic rationale supporting their therapeutic use, critically evaluate the clinical evidence across individual dermatologic conditions, compare the pharmacological characteristics of the three currently available topical analogs, and discuss practical considerations, current limitations, and future research priorities. By integrating molecular mechanisms with clinical evidence, this review aims to provide a practical framework to guide both future research and the rational clinical application of topical vitamin D analogs beyond psoriasis [1,2,15,16].

## 2. Literature Search Strategy

A structured literature search was conducted in PubMed/MEDLINE, Scopus, Embase, and Web of Science for relevant studies published up to September 2026. Search terms included combinations of “vitamin D”, “vitamin D analogs”, “calcipotriol/calcipotriene”, “calcitriol”, “tacalcitol”, “topical”, “cutaneous”, “drug delivery”, and condition-specific terms including “acne”, “atopic dermatitis/eczema”, “vitiligo”, “basal cell carcinoma”, “actinic keratosis”, “morphea/localized scleroderma”, “ichthyosis”, “alopecia areata”, and “lichen planus”. No formal language restriction was applied; however, the final evidence base consisted predominantly of English-language publications. Studies evaluating topical vitamin D analogs in dermatological conditions other than psoriasis were considered for inclusion, including randomized and comparative trials, observational studies, case series, and case reports. Preclinical studies were included when relevant to pharmacology, cutaneous pharmacokinetics, or drug-delivery systems, while reviews, meta-analyses, and guidelines were used for contextualization and identification of relevant primary studies. Publications unrelated to topical or cutaneous administration were excluded. Given the narrative nature and heterogeneity of the evidence, no formal meta-analysis or standardized risk-of-bias assessment was performed. Conditions were selected for detailed discussion based on available human clinical evidence and/or a relevant mechanistic rationale. Conditions supported mainly by isolated reports or preclinical evidence were considered as emerging applications. Actinic keratosis was retained as adjacent clinical evidence rather than developed as a separate disease-specific subsection because the available clinical evidence predominantly evaluates calcipotriol as part of a specific combination regimen with 5-fluorouracil and therefore does not establish an independent therapeutic role for topical vitamin D analogs in this condition. Other reported off-label dermatological applications supported mainly by isolated clinical reports or preliminary or preclinical evidence were considered under emerging applications rather than as separate disease-specific sections. Psoriasis was excluded because it represents an established indication and falls outside the scope of this review. Evidence was classified according to study design and methodological strength, with greater weight assigned to systematic reviews/meta-analyses and randomized controlled trials, followed by comparative or observational studies, uncontrolled studies, case series, and case reports. Preclinical evidence was considered supportive of biological plausibility rather than evidence of clinical efficacy.

## 3. Biology and Pharmacology of Topical Vitamin D Analogs

### 3.1. Molecular Structure

**Figure 2:**
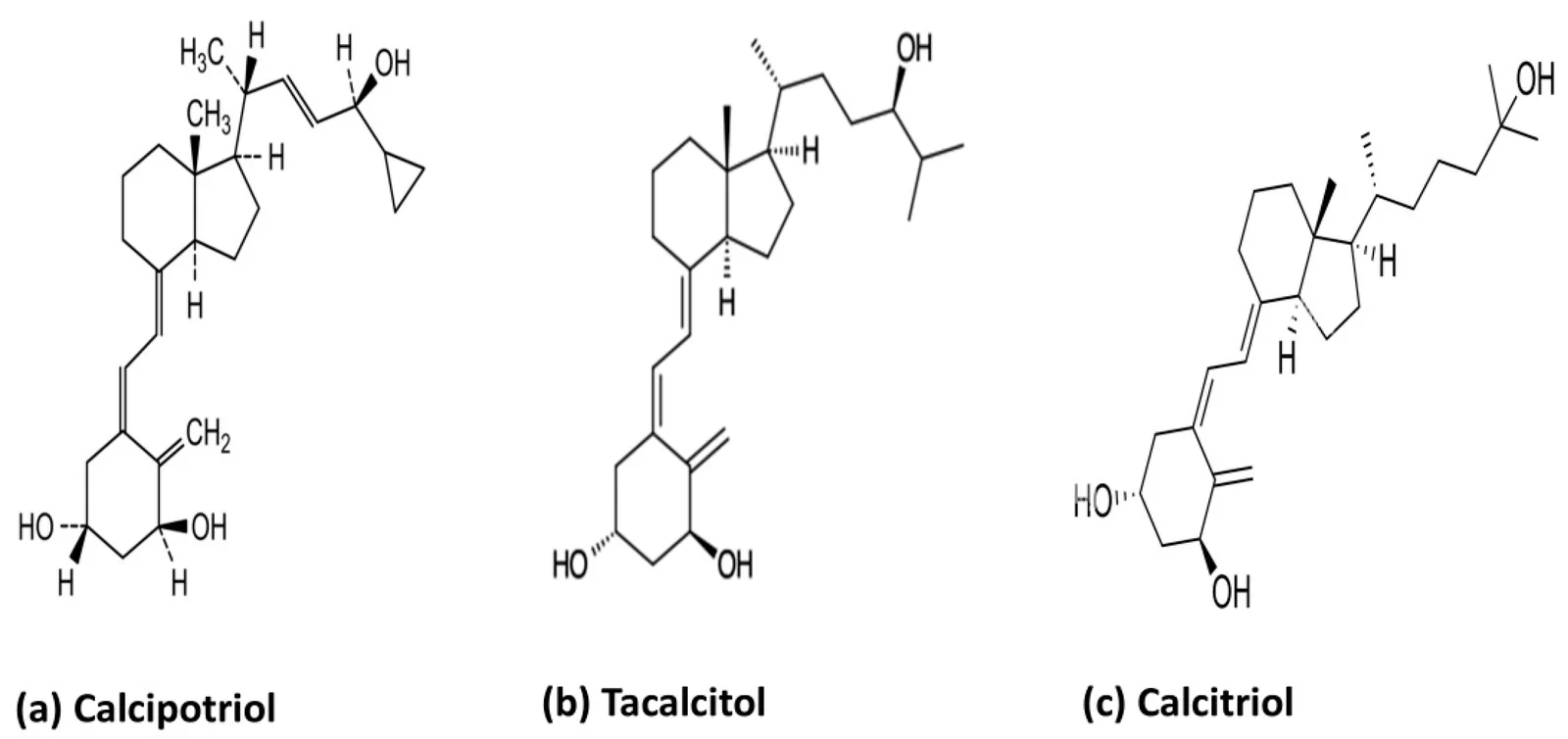
Chemical structures of calcitriol, calcipotriol, and tacalcitol.

Calcitriol (1,25-dihydroxyvitamin D_3_) is the biologically active form of vitamin D_3_ and serves as the structural and pharmacological reference for synthetic vitamin D analogs. Calcipotriol and tacalcitol retain the characteristic secosteroid scaffold of calcitriol but incorporate structural modifications designed to preserve local VDR-mediated activity while modifying metabolic stability and reducing systemic calcemic effects [4,5]. Calcipotriol contains modifications in the side chain that alter its metabolic profile and contribute to rapid systemic inactivation, whereas tacalcitol is a hydroxylated vitamin D_3_ derivative with high VDR activity and favorable topical tolerability [4,5,17]. These structural differences contribute to variations in pharmacological behavior among the three compounds and provide the basis for their different therapeutic and safety profiles following topical administrations illustrated in Figure 2.

### 3.2. Pharmacological Characteristics

Despite sharing a common mechanism of action, calcipotriol, tacalcitol, and calcitriol demonstrate clinically relevant pharmacological differences. Calcipotriol is the most extensively investigated topical vitamin D analog and accounts for the majority of clinical evidence concerning both approved and off-label dermatologic applications. It exhibits high VDR affinity while undergoing rapid hepatic metabolism, resulting in minimal systemic calcium effects despite potent local biological activity. Its favorable balance between efficacy and safety explains its predominance within the clinical literature [4,5]. Tacalcitol possesses prolonged receptor occupancy and greater metabolic stability than calcipotriol, permitting once-daily administration. Clinical studies in psoriasis have reported excellent local tolerability, making tacalcitol particularly attractive for long-term therapy, although considerably fewer studies have evaluated its use in non-psoriatic skin diseases [17]. Calcitriol, the naturally active form of vitamin D_3_, demonstrates lower irritancy than synthetic analogs and is generally preferred for sensitive anatomical sites, including the face, flexural regions, and intertriginous skin [18]. However, because systemic absorption of calcitriol has a greater potential to influence calcium metabolism, prolonged treatment of extensive body surface areas requires greater caution than treatment with synthetic analogs [4,19]. Although these pharmacological differences are well recognized, virtually no randomized studies have directly compared the three analogs in non-psoriatic skin diseases. Consequently, current therapeutic selection relies primarily on extrapolation from psoriasis studies, clinician experience, and anatomical considerations rather than comparative evidence [4,5,17,18,20]. The principal pharmacological and pharmaceutical characteristics of calcipotriol, tacalcitol, and calcitriol are summarized in Table 1.

### 3.3. Pharmacodynamics and Vitamin D Receptor Signaling

The pharmacological activity of topical vitamin D analogs is mediated primarily through activation of the vitamin D receptor (VDR), a member of the nuclear receptor superfamily that functions as a ligand-activated transcription factor. Following topical application and penetration into viable epidermal layers, vitamin D analogs bind to intracellular VDR expressed in keratinocytes, melanocytes, sebocytes, dermal fibroblasts, and multiple immune cell populations. Ligand binding induces a conformational change in the receptor, facilitating heterodimerization with the retinoid X receptor (RXR). The activated VDR–RXR complex subsequently translocates to the nucleus, where it recognizes vitamin D response elements (VDREs) within promoter regions of target genes, thereby regulating transcriptional programs involved in epidermal homeostasis, cellular differentiation, immune regulation, and tissue remodeling [2,3,22]. Genomic activation of VDR influences the expression of hundreds of genes involved in skin physiology. In keratinocytes, vitamin D signaling suppresses excessive proliferation while promoting terminal differentiation through increased expression of structural proteins such as involucrin, loricrin, filaggrin, and transglutaminase. Simultaneously, VDR activation regulates genes controlling cell-cycle progression, apoptosis, and maintenance of epidermal barrier integrity. These coordinated effects restore physiological epidermal architecture while limiting hyperproliferative responses that characterize several inflammatory and inherited skin disorders [3,23]. Beyond its effects on epidermal differentiation, VDR activation exerts profound immunomodulatory activity. Vitamin D signaling suppresses pro-inflammatory T-helper 1 (Th1) and T-helper 17 (Th17) immune responses while promoting regulatory T-cell differentiation and anti-inflammatory cytokine production. In parallel, antigen presentation by dendritic cells is attenuated, resulting in reduced production of inflammatory mediators including interleukin (IL)-2, IL-6, IL-17, IL-23, interferon-γ, and tumor necrosis factor-α. These immunological effects provide a mechanistic basis for the investigation of topical vitamin D analogs in immune-mediated skin disorders such as vitiligo, morphea, alopecia areata, and atopic dermatitis [3,24,25,26,27,28]. Vitamin D signaling also enhances innate immune function through transcriptional regulation of antimicrobial peptides, particularly cathelicidin (LL-37) and β-defensins. These peptides contribute not only to antimicrobial defense against bacterial, viral, and fungal pathogens but also to wound repair, immune surveillance, and maintenance of cutaneous barrier function. Such effects are particularly relevant in acne vulgaris, where modulation of host inflammatory responses may complement conventional antimicrobial therapy [29,30,31,32]. Accumulating evidence further suggests that vitamin D analogs may induce rapid non-genomic responses independent of direct gene transcription. These effects have been associated with modulation of intracellular calcium signaling, activation of phospholipase C, protein kinase C, mitogen-activated protein kinase (MAPK), and phosphatidylinositol-3 kinase (PI3K)/Akt pathways. Although the precise contribution of these rapid signaling mechanisms to the clinical efficacy of topical vitamin D analogs remains incompletely understood, they are believed to complement classical genomic actions by influencing cellular migration, survival, and inflammatory responses [25,33]. Importantly, the widespread distribution of VDR throughout the skin explains the broad therapeutic potential of topical vitamin D analogs. In addition to keratinocytes, functional VDR expression has been demonstrated in melanocytes, sebocytes, dermal fibroblasts, dendritic cells, macrophages, mast cells, endothelial cells, and activated T lymphocytes. Consequently, activation of a single receptor system simultaneously influences multiple interconnected biological pathways involved in epidermal differentiation, pigmentation, innate and adaptive immunity, fibrosis, wound healing, and tumor biology. This pleiotropic pharmacodynamic profile distinguishes topical vitamin D analogs from therapies targeting individual inflammatory mediators and provides the biological basis for their therapeutic repurposing across a wide spectrum of non-psoriatic dermatologic disorders [1,2,3,20].

### 3.4. Pharmacokinetics of Topical Vitamin D Analogs

**Figure 3:**
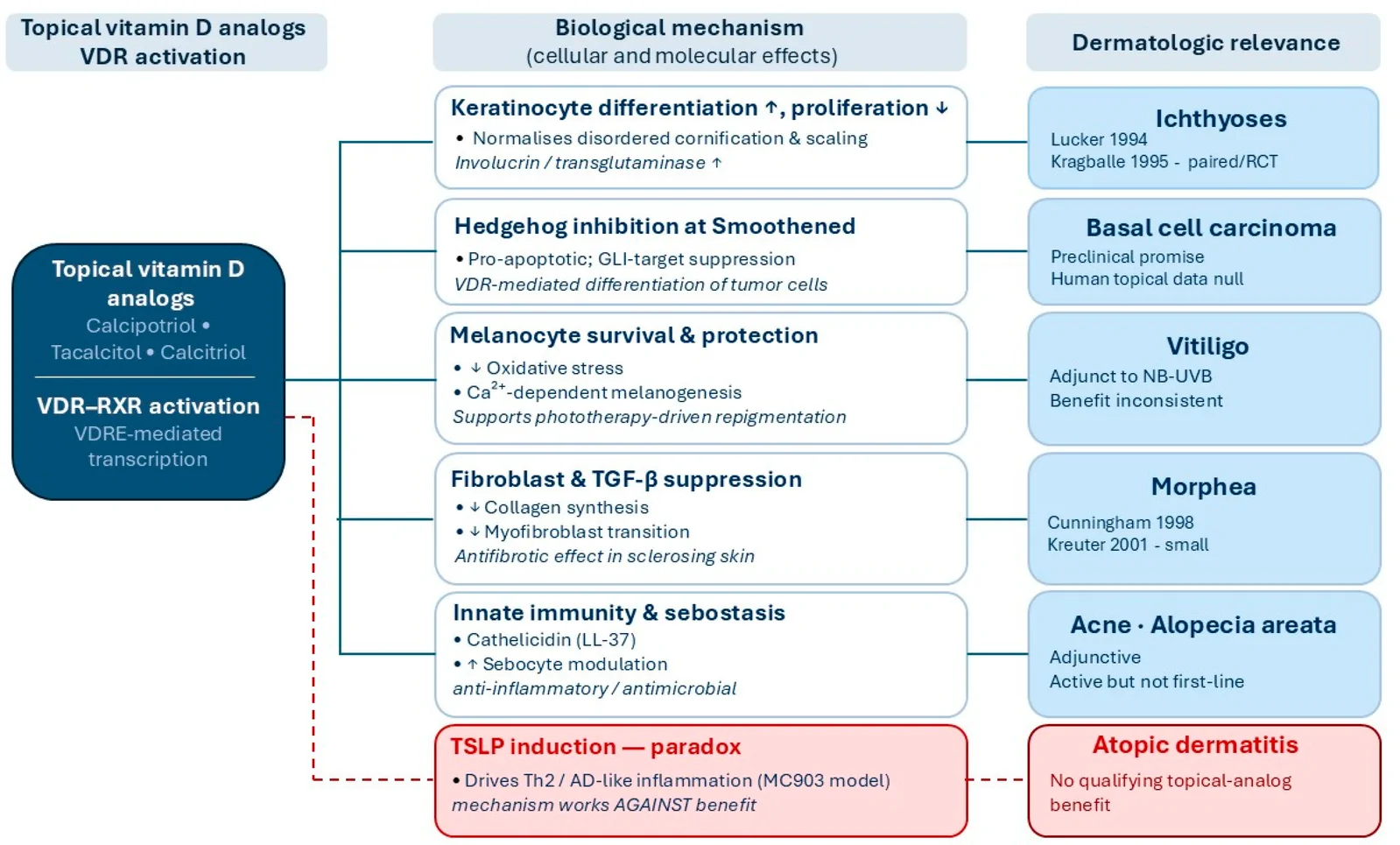
Mechanisms linking activation of the vitamin D receptor (VDR) to the therapeutic effects of topical vitamin D analogs in non-psoriatic skin diseases. Ligand binding activates the VDR/RXR complex, regulating lineage-specific pathways involved in epidermal differentiation, immune modulation, melanocyte biology, fibrosis, and tumor suppression. Solid line = therapeutic benefit; dashed red line = counter-therapeutic pathway.

Following topical administration, vitamin D analogs penetrate the stratum corneum and diffuse into the viable epidermis, where they exert predominantly local pharmacological effects through activation of the vitamin D receptor. Their cutaneous bioavailability is influenced by several factors, including the physicochemical properties of the individual analog, formulation type, anatomical site of application, skin barrier integrity, and the extent of the treated body surface area. Under recommended therapeutic conditions, systemic absorption is generally low, allowing high local efficacy while minimizing alterations in calcium homeostasis. Nevertheless, prolonged treatment of extensive skin surfaces or use under occlusive conditions may increase systemic exposure, particularly with calcitriol, necessitating careful consideration of dose and duration in susceptible patients. Differences in metabolic stability and calcemic activity among calcipotriol, tacalcitol, and calcitriol further contribute to their distinct safety profiles and clinical applications [4,5,17,21] while the principal VDR-mediated pathways relevant to their therapeutic effects are summarized in Figure 3.

Available quantitative pharmacokinetic data indicate differences among individual topical vitamin D compounds, although direct comparative studies remain limited. For calcipotriol under recommended therapeutic conditions, systemic absorption is generally limited, favoring predominantly local exposure while reducing the potential for systemic effects on calcium homeostasis. In a small radiolabel study in patients with psoriasis treated with calcipotriol 50 μg/g ointment, less than 1% of the applied dose was estimated to be absorbed [10]. Experimental permeation studies further indicate substantial cutaneous retention of calcipotriol, with approximately 40% of the applied dose retained in porcine skin and approximately 60% in rat and mouse skin after 20 h, while transdermal permeation remained limited [34]. Tacalcitol also demonstrates low systemic exposure, with less than 0.5% of the applied dose reported to be systemically absorbed through psoriatic skin [12]. Following absorption, tacalcitol binds to vitamin D-binding protein and is metabolized principally to 1α,24,25-trihydroxyvitamin D_3_, a metabolite with lower vitamin D activity than the parent compound [12]. In contrast, mean percutaneous absorption of topical calcitriol has been estimated at approximately 10%, with unchanged calcitriol and metabolites detectable systemically [11]. However, differences in study design, formulation, treated skin, and analytical methodology preclude direct quantitative comparison among these compounds.

Cutaneous disposition is also influenced by vehicle composition, skin-barrier integrity, and application conditions. Occlusion can increase penetration through the stratum corneum and has been reported to enhance the clinical response to topical calcipotriol [35]; however, because enhanced penetration may increase systemic exposure, occlusive application is discouraged in current UK product information for some vitamin D formulations [10,11]. Importantly, most quantitative pharmacokinetic data for topical vitamin D compounds have been obtained in psoriatic, healthy, or experimental skin rather than in the non-psoriatic dermatological conditions considered in this review. Compound-specific information on cutaneous retention, systemic exposure, and the influence of disease-related barrier alterations in acne, atopic dermatitis, vitiligo, morphea, ichthyosis, and alopecia areata remains scarce. Consequently, pharmacokinetic findings derived from psoriatic or experimental skin should not be assumed to directly represent drug disposition in non-psoriatic diseased skin.

### 3.5. Pharmaceutical Formulations and Topical Drug Delivery

The clinical performance of topical vitamin D analogs depends not only on their pharmacological activity but also on the characteristics of the delivery system. Commercially available formulations include ointments, creams, gels, foams, and scalp solutions, each designed to optimize cutaneous drug delivery while minimizing systemic exposure. Formulation selection influences drug release, penetration through the stratum corneum, patient acceptability, and treatment adherence, all of which may ultimately affect therapeutic outcomes [6,7,8,9]. Recent advances in topical drug-delivery research have explored liposomes, nanoemulsions, solid lipid nanoparticles (SLNs), nanostructured lipid carriers (NLCs), polymeric nanocarriers, and microneedle-assisted systems as strategies to improve drug stability, controlled release, cutaneous retention, and skin targeting [6,7,8,9].

In particular, advanced delivery systems have been studied experimentally for transdermal administration of vitamin D analogs, mainly calcipotriol. Liposomes have been considered as vehicles for calcipotriol, calcitriol, and tacalcitol, and experimental studies confirmed the effect of vesicle formulation on skin deposition and local pharmacology of drugs owing to their composition, size, membrane properties, and PEGylation [36,37]. Other lipid nanovehicles such as nanoemulsions, nanostructured lipid carriers, and solid lipid nanoparticles have been developed for calcipotriol alone or in combination with other topical agents. Enhanced cutaneous penetration and pharmacological activity were shown experimentally with nanoemulsion formulations of calcipotriol, while SLNs and NLCs have been used in experimental ex vivo permeation studies in order to facilitate drug localization in the epidermis and dermis, as well as prevent its diffusion into the receptor phase [38,39,40,41]. Pickering nanoemulsions constitute an additional approach to enhance calcipotriol delivery [42]. In more recent studies, nanosuspensions incorporated into dissolving microneedle arrays have been proposed as vehicles to overcome the stratum corneum barrier and deliver the drug locally into the intradermal space, with comparable anti-psoriatic activity in preclinical studies to conventional topical calcipotriol therapy [43]. These data indicate growing interest from the pharmaceutical industry in improving the transdermal delivery of vitamin D analogs; however, the majority of the evidence base is preclinical, oriented to calcipotriol and psoriasis, and has not yet provided any advantages over conventional formulations..The main advanced cutaneous delivery systems investigated for vitamin D compounds and their experimental characteristics are summarized in Table 2.

### 3.6. Safety Considerations and Clinical Precautions

Topical vitamin D analogs are generally well tolerated when used within recommended exposure limits; however, local irritation and disturbances of calcium metabolism represent the principal safety considerations. Local adverse reactions may include erythema, pruritus, burning, dryness, and dermatitis. Recommendations for use on sensitive anatomical sites differ among compounds and formulations. For example, according to current UK product information, calcipotriol should not be applied to the face because of the risk of irritation, whereas calcitriol may be applied to the face with caution and tacalcitol may be used on the face and flexural areas [10,11,12]. Systemic disturbances of calcium metabolism are uncommon at recommended topical doses but may occur with excessive exposure. Hypercalcemia and hypercalciuria have been reported with calcipotriol, while excessive calcitriol exposure may increase serum and urinary calcium concentrations [10,11]. Occlusion may enhance cutaneous penetration and is discouraged in UK product information for calcipotriol and calcitriol because of the potential effects on calcium metabolism [10,11]. Particular caution is warranted in patients with pre-existing hypercalcemia, disorders of calcium metabolism, or renal impairment. For tacalcitol, monitoring of serum calcium is specifically recommended in patients at risk of hypercalcemia and in those with renal impairment [12].

Primary clinical evidence supports a relationship between extensive topical calcipotriol exposure and disturbances in calcium homeostasis. In a controlled study of 16 patients with extensive plaque psoriasis treated with high-dose topical calcipotriol (50 μg/g; up to 360 g/week for 2 weeks), five patients developed hypercalcemia, accompanied by dose-dependent increases in serum and urinary calcium and suppression of parathyroid hormone [44]. In addition, a hypercalcemic crisis has been reported in a 17-year-old patient following excessive topical calcipotriol use for ichthyosis [45].

Maximum recommended exposure is compound- and formulation-specific. According to current UK product information, calcipotriol 50 μg/g ointment should not exceed 100 g/week, corresponding to a maximum total calcipotriol exposure of 5 mg/week when multiple calcipotriol-containing formulations are used [10]. For calcitriol 3 μg/g ointment, treatment should not exceed 35% of the body surface area or 30 g/day [11], whereas tacalcitol 4 μg/g ointment should not exceed 10 g/day [12]. These limits should not be extrapolated across different formulations or jurisdictions.

Special populations require additional consideration. Pediatric recommendations differ among products, and long-term safety data remain limited. Pregnancy data are also insufficient; according to current UK product information, calcipotriol should be avoided as a precaution, calcitriol should be used only when clearly necessary, and tacalcitol should be avoided unless no safer alternative is available [10,11,12]. Recommendations regarding prolonged treatment are likewise product-specific. Therefore, pediatric use, pregnancy, prolonged therapy, treatment of extensive body surface areas, and use in patients with renal impairment or disorders of calcium metabolism should be guided by the prescribing information applicable to the specific compound, formulation, and jurisdiction.

### 3.7. Biological Effects Relevant to Non-Psoriatic Skin Diseases

#### 3.7.1. Regulation of Keratinocyte Proliferation and Differentiation

One of the best-characterized biological functions of vitamin D signaling is the regulation of epidermal homeostasis. Activation of the vitamin D receptor suppresses excessive keratinocyte proliferation while simultaneously promoting terminal differentiation through coordinated regulation of genes involved in cell-cycle control and epidermal maturation. These effects include increased expression of structural proteins such as loricrin, filaggrin, and transglutaminase, together with inhibition of hyperproliferative signaling pathways. Consequently, vitamin D analogs contribute to restoration of epidermal architecture while preserving normal barrier function [23]. Although these mechanisms were originally characterized in psoriasis, they are equally relevant to several non-psoriatic disorders characterized by abnormal epidermal differentiation. In inherited ichthyoses, impaired terminal differentiation represents a central pathogenic event, providing a direct biological rationale for topical vitamin D therapy. Similarly, restoration of physiological differentiation may contribute to limiting uncontrolled keratinocyte proliferation in certain premalignant and neoplastic skin conditions, including basal cell carcinoma. Thus, regulation of keratinocyte differentiation represents one of the fundamental mechanisms through which topical vitamin D analogs may exert therapeutic activity beyond psoriasis [23,46,47].

#### 3.7.2. Immunomodulatory Effects

The immunomodulatory properties of vitamin D analogs extend far beyond suppression of epidermal hyperproliferation and represent one of the principal reasons for their investigation in inflammatory skin diseases. Activation of the VDR influences both innate and adaptive immune responses by modulating cytokine production, antigen presentation, and lymphocyte differentiation. Collectively, these actions promote restoration of immune homeostasis while limiting excessive inflammatory activation [3,24]. Vitamin D signaling suppresses pro-inflammatory T-helper 1 (Th1) and T-helper 17 (Th17) responses while promoting the expansion and functional activity of regulatory T cells (Tregs), thereby contributing to immune tolerance. Simultaneously, production of inflammatory mediators including interleukin (IL)-2, IL-6, IL-17, IL-23, interferon-γ, and tumor necrosis factor-α (TNF-α) is reduced, whereas anti-inflammatory cytokines such as IL-10 may be enhanced. These coordinated effects provide a strong biological rationale for investigating topical vitamin D analogs in disorders characterized by dysregulated immune activation, including atopic dermatitis, vitiligo, morphea, and alopecia areata [3,22,23,27,28]. These findings indicate that vitamin D/VDR signaling can modulate immune responses through effects on T-cell differentiation, cytokine production, and antigen-presenting cells [3,24,25]. However, these mechanistic observations should not be interpreted as evidence that topical vitamin D analogs provide superior immune regulation compared with conventional topical anti-inflammatory therapies.

Nevertheless, the extent to which these immunological mechanisms translate into clinically meaningful therapeutic benefit varies substantially among different dermatologic conditions, highlighting the importance of disease-specific pathogenic pathways.

#### 3.7.3. Regulation of Innate Immunity and Antimicrobial Peptides

Beyond its effects on adaptive immunity, vitamin D signaling plays a fundamental role in regulating the skin’s innate immune defense. Activation of the vitamin D receptor stimulates the transcription of several antimicrobial peptides (AMPs), most notably cathelicidin (LL-37) and β-defensins, which constitute essential components of the cutaneous antimicrobial barrier. These peptides exhibit broad-spectrum activity against bacteria, viruses, and fungi while also participating in immune surveillance, wound healing, and maintenance of epidermal integrity [29,30,31]. The ability of vitamin D analogs to modulate antimicrobial peptide expression has attracted particular interest in acne vulgaris, where dysregulated innate immune responses contribute substantially to disease pathogenesis. Rather than acting solely through direct antimicrobial activity against *Cutibacterium acnes* (*C. acnes*), LL-37 influences chemotaxis, cytokine production, angiogenesis, and tissue repair, suggesting that vitamin D signaling may help restore immune homeostasis within the pilosebaceous unit. Experimental studies further demonstrate that vitamin D analogs suppress inflammatory cytokines, including IL-6, IL-8, and TNF-α, in stimulated sebocytes, supporting a combined antimicrobial and anti-inflammatory mechanism of action [30,31,32]. These observations suggest that topical vitamin D analogs may complement, rather than replace, conventional antimicrobial therapies by targeting the host immune response instead of bacterial proliferation alone. Such an approach may become increasingly relevant as concerns regarding antimicrobial resistance continue to influence acne management and encourage the development of non-antibiotic therapeutic strategies.

#### 3.7.4. Effects on Melanocyte Biology and Oxidative Stress

Vitamin D signaling also plays an important role in maintaining melanocyte homeostasis. Melanocytes express functional vitamin D receptors, and activation of these receptors regulates multiple biological processes, including melanocyte survival, differentiation, melanogenesis, and resistance to oxidative stress. Given that oxidative injury and autoimmune destruction are central features of vitiligo pathogenesis, these observations provide a strong biological rationale for investigating topical vitamin D analogs as adjunctive therapies in pigmentary disorders [48,49]. Experimental studies have demonstrated that vitamin D enhances melanocyte survival by reducing oxidative stress-induced apoptosis and modulating Wnt/β-catenin signaling, a pathway closely associated with melanocyte regeneration and pigmentation. In parallel, the immunomodulatory actions of vitamin D may attenuate the cytotoxic T-cell responses responsible for melanocyte destruction. These complementary mechanisms are believed to underlie the synergistic effects observed when topical vitamin D analogs are combined with narrowband ultraviolet B (NB-UVB) phototherapy, which stimulates melanocyte proliferation and migration while vitamin D signaling promotes melanocyte survival and stabilization [48,49,50,51]. Although the precise molecular interactions remain incompletely understood, the combined effects on oxidative stress, immune regulation, and melanocyte biology distinguish vitamin D analogs from conventional anti-inflammatory topical therapies and support their investigation as biologically targeted adjuncts for vitiligo management.

#### 3.7.5. Antifibrotic Activity

Beyond their immunomodulatory properties, topical vitamin D analogs exhibit antifibrotic activity through direct effects on dermal fibroblasts and extracellular matrix remodeling. Dermal fibroblasts express functional vitamin D receptors, and activation of VDR signaling suppresses fibroblast proliferation, inhibits myofibroblast differentiation, and reduces excessive collagen synthesis. These effects are mediated primarily through modulation of the transforming growth factor-beta (TGF-β)/SMAD signaling pathway, a central regulator of tissue fibrosis in both cutaneous and systemic fibrotic disorders [52,53,54]. Experimental studies have demonstrated that vitamin D signaling downregulates the expression of profibrotic mediators while attenuating extracellular matrix deposition and fibroblast activation. In addition to these direct effects, the anti-inflammatory properties of vitamin D may further limit fibrosis by reducing the chronic inflammatory stimuli that drive persistent tissue remodeling. Collectively, these mechanisms provide a strong biological rationale for investigating topical vitamin D analogs in localized scleroderma (morphea), where excessive collagen deposition and progressive dermal sclerosis represent the principal pathological features [53,54,55]. Although clinical evidence remains limited, the mechanistic consistency between VDR signaling and established profibrotic pathways suggests that topical vitamin D analogs may represent valuable adjunctive therapies, particularly during the inflammatory phase of disease when fibroblast activation remains potentially reversible.

## 4. Clinical Applications of Topical Vitamin D Analogs Beyond Psoriasis

### 4.1. Acne Vulgaris

#### 4.1.1. Disease Background and Biological Rationale

Acne vulgaris is one of the most prevalent inflammatory skin disorders worldwide, affecting approximately 85% of adolescents and a substantial proportion of adults. Its pathogenesis is multifactorial, involving excessive sebum production, follicular hyperkeratinization, colonization by *C. acnes*, and a dysregulated innate and adaptive immune response. While current therapies, including topical retinoids, benzoyl peroxide, antibiotics, and hormonal agents, are generally effective, treatment failure, antibiotic resistance, local irritation, and poor adherence remain important clinical challenges. Consequently, considerable interest has emerged in identifying adjunctive therapies capable of targeting multiple pathogenic mechanisms simultaneously [30,31,32,56]. The biological rationale for topical vitamin D analogs in acne extends beyond their established effects on keratinocyte differentiation. Sebocytes express functional vitamin D receptors, allowing direct regulation of sebocyte proliferation, differentiation, and lipid metabolism. Experimental studies have further demonstrated that vitamin D signaling suppresses the production of pro-inflammatory cytokines, including IL-6, IL-8, and TNF-α, while stimulating antimicrobial peptide expression, particularly cathelicidin (LL-37), thereby enhancing innate immune defense against *C. acnes*. Collectively, these observations suggest that topical vitamin D analogs may simultaneously modulate follicular hyperkeratinization, sebaceous gland activity, and cutaneous inflammation, providing a biologically plausible basis for therapeutic repurposing in acne vulgaris [30,31,32].

#### 4.1.2. Clinical Evidence

Clinical evaluation of topical vitamin D analogs in acne vulgaris remains limited, with available evidence consisting primarily of small randomized trials and comparative split-face studies. Nevertheless, the published literature consistently suggests that calcipotriol possesses measurable anti-inflammatory activity, although its clinical efficacy appears inferior to that of established topical retinoids. The strongest evidence derives from a triple-blind, randomized, split-face clinical trial involving 60 patients, in which once-daily calcipotriol 0.005% cream significantly reduced both inflammatory and non-inflammatory acne lesions compared with placebo over eight weeks. However, adapalene 0.1% gel produced significantly greater clinical improvement and higher patient satisfaction, indicating that although calcipotriol demonstrates biological activity, it is unlikely to replace first-line topical retinoids [57]. These findings are supported by an earlier prospective split-face study involving 40 patients, which similarly demonstrated significant reductions in acne lesion counts following treatment with calcipotriol. Importantly, both studies suggest that vitamin D analogs possess clinically relevant anti-inflammatory effects while exhibiting acceptable local tolerability, supporting their potential role as adjunctive rather than primary therapeutic agents [58]. Although combination approaches targeting complementary pathogenic pathways are biologically attractive, robust randomized studies evaluating the additive clinical benefit of vitamin D analogs within multimodal acne regimens remain unavailable. From a drug-delivery perspective, the available clinical studies primarily investigated calcipotriol 0.005% in a cream formulation, applied once daily to facial lesions, providing a relatively non-occlusive vehicle suitable for acne-prone skin [57,58].

#### 4.1.3. Critical Appraisal

Although the biological rationale supporting topical vitamin D analogs in acne vulgaris is compelling, current clinical evidence remains insufficient to support routine therapeutic use. Existing studies are limited by small sample sizes, short follow-up periods, heterogeneous outcome measures, and the absence of direct comparisons among different vitamin D analogs. Furthermore, nearly all available trials have evaluated calcipotriol, leaving the potential roles of tacalcitol and calcitriol largely unexplored. From a clinical perspective, topical vitamin D analogs should currently be viewed as investigational adjunctive therapies rather than alternatives to established acne treatments. Their greatest potential may lie in combination regimens that reduce reliance on topical antibiotics or improve inflammatory control in selected patients. Given increasing concerns regarding antimicrobial resistance, future randomized controlled trials evaluating vitamin D analogs within multimodal acne treatment strategies appear justified.

#### 4.1.4. Future Perspectives

Future research should prioritize adequately powered randomized controlled trials comparing different vitamin D analogs both as monotherapy and in combination with established acne treatments. Standardized clinical endpoints, longer follow-up periods, and biomarker-based patient stratification may help identify individuals most likely to benefit from VDR-targeted therapy. Further investigation into the interaction between vitamin D signaling, sebocyte biology, and the cutaneous microbiome may also provide new opportunities for personalized acne management.

### 4.2. Atopic Dermatitis

#### 4.2.1. Disease Background and Biological Rationale

Atopic dermatitis (AD) is a chronic inflammatory skin disease characterized by epidermal barrier dysfunction, immune dysregulation, and recurrent eczematous lesions. The pathogenesis of AD involves a complex interplay between genetic susceptibility, impaired skin barrier integrity, altered cutaneous microbiota, and a predominantly T-helper 2 (Th2)-driven immune response, particularly during the acute phase of disease. Current treatment strategies rely primarily on emollients, topical corticosteroids, topical calcineurin inhibitors, phosphodiesterase-4 inhibitors, and, in moderate-to-severe disease, targeted biologic therapies and Janus kinase (JAK) inhibitors. Despite these advances, long-term disease control remains challenging for many patients because of recurrent flares, treatment-related adverse effects, and the need for prolonged therapy [59,60,61,62]. The biological rationale for investigating topical vitamin D analogs in AD is supported by several mechanistic observations. Vitamin D signaling contributes to epidermal barrier maintenance through regulation of keratinocyte differentiation and increased expression of structural proteins, including filaggrin, involucrin, and loricrin. In addition, activation of the vitamin D receptor promotes antimicrobial peptide production, enhances innate immune defense, and modulates adaptive immune responses by suppressing pro-inflammatory cytokine production while promoting regulatory T-cell activity. Collectively, these effects suggest that vitamin D analogs could theoretically improve both barrier function and immune homeostasis in patients with AD [1,2,59,60].

#### 4.2.2. Clinical Evidence

Direct clinical evidence supporting topical vitamin D analogs in classic atopic dermatitis is currently lacking. The available human evidence derives primarily from chronic hand eczema, which is clinically distinct from atopic dermatitis and is therefore considered here as indirect or adjacent evidence rather than direct evidence of efficacy in atopic dermatitis. As adjacent clinical evidence, one comparative clinical study reported that topical calcipotriol achieved improvements comparable to those of a topical corticosteroid in chronic hand eczema. Although encouraging, these findings cannot be directly extrapolated to AD because chronic hand eczema represents a clinically distinct disorder with a different pathophysiological profile. Furthermore, no studies have established superiority over standard anti-inflammatory therapies or demonstrated additional benefit when vitamin D analogs are combined with established topical treatments [63]. Experimental studies have also produced conflicting findings. While vitamin D signaling promotes epidermal barrier repair and immune regulation, topical calcipotriol has been shown to induce thymic stromal lymphopoietin (TSLP) expression and an AD-like inflammatory phenotype in murine models [64,65]. These observations suggest that the biological effects of vitamin D analogs may differ substantially between healthy and inflamed skin, complicating translation from experimental models to clinical practice [50,54]. The available clinical evidence has predominantly evaluated calcipotriol in an ointment formulation, providing an occlusive vehicle that may enhance stratum corneum hydration and local drug retention in dry and barrier-impaired skin [63].

#### 4.2.3. Critical Appraisal

Among all dermatologic conditions reviewed, atopic dermatitis presents one of the greatest discrepancies between mechanistic plausibility and clinical evidence. Multiple biological pathways support a potential therapeutic role for vitamin D analogs, including enhancement of epidermal barrier function, modulation of adaptive immunity, and stimulation of innate antimicrobial defenses. Nevertheless, these promising mechanisms have not translated into convincing clinical efficacy. Several factors may contribute to this discrepancy. First, the pro-inflammatory effects observed in experimental models raise concerns regarding local irritation and disease exacerbation in barrier-disrupted skin. Second, increased percutaneous absorption in AD may narrow the therapeutic window and increase the likelihood of adverse local reactions. Finally, the absence of adequately designed randomized clinical trials makes it impossible to determine whether the limited clinical use of topical vitamin D analogs reflects genuine therapeutic failure or simply insufficient investigation. Consequently, current evidence does not support routine use of topical vitamin D analogs in atopic dermatitis. At present, these agents should be regarded as investigational therapies whose potential remains biologically intriguing but clinically unproven.

#### 4.2.4. Future Perspectives

Future research should focus on identifying patient subgroups most likely to benefit from topical vitamin D analog therapy, particularly individuals with specific barrier defects or altered VDR signaling. Carefully designed randomized controlled trials evaluating optimized formulations, lower concentrations, or combination regimens with established topical anti-inflammatory agents may clarify whether the favorable mechanistic profile of vitamin D analogs can be translated into meaningful clinical benefit. In parallel, further investigation into the interaction between vitamin D signaling, epidermal barrier biology, and TSLP-mediated inflammation may help explain the apparent disconnect between experimental findings and clinical outcomes.

### 4.3. Vitiligo

#### 4.3.1. Disease Background and Biological Rationale

Vitiligo is a chronic acquired depigmenting disorder characterized by the selective destruction of epidermal melanocytes, resulting in well-demarcated depigmented macules and patches. Although the precise etiology remains incompletely understood, current evidence supports a multifactorial pathogenesis involving autoimmune mechanisms, oxidative stress, genetic susceptibility, and impaired melanocyte regeneration. Narrowband ultraviolet B (NB-UVB) phototherapy remains the cornerstone of treatment for generalized vitiligo, whereas topical corticosteroids and calcineurin inhibitors are recommended for localized disease. Nevertheless, treatment responses remain variable, and complete repigmentation is achieved in only a subset of patients, highlighting the need for effective adjunctive therapies [48,49,51]. The biological rationale for topical vitamin D analogs in vitiligo is supported by several complementary mechanisms. Functional VDR expression in melanocytes enables vitamin D signaling to regulate melanocyte proliferation, differentiation, and survival while reducing oxidative stress-induced apoptosis. In addition, vitamin D modulates local immune responses by suppressing autoreactive T-cell activity and promoting regulatory immune pathways, thereby addressing two fundamental components of vitiligo pathogenesis. These combined effects provide a strong mechanistic basis for combining topical vitamin D analogs with phototherapy, which primarily stimulates melanocyte proliferation and migration [48,49,50,51].

#### 4.3.2. Clinical Evidence

Among the non-psoriatic indications reviewed, vitiligo has one of the most extensively studied clinical evidence bases for the use of topical vitamin D analogs. Clinical investigations have primarily evaluated these agents on their use as adjunctive therapies rather than monotherapy, particularly in combination with NB-UVB phototherapy and psoralen plus ultraviolet A (PUVA). The most comprehensive evidence derives from a 2022 systematic review and meta-analysis including 14 clinical studies and 642 participants [51]. The effect of adding topical vitamin D analogs differed according to phototherapy modality. When combined with NB-UVB, calcipotriol or tacalcitol increased the proportion of patients achieving ≥50% repigmentation compared with NB-UVB alone (RR 1.67, 95% CI 1.21–2.31). Combination therapy was also associated with a lower risk of treatment failure (RR 0.43, 95% CI 0.22–0.85) and a higher probability of an excellent response (RR 7.48, 95% CI 1.09–51.13), although the wide confidence interval for the latter indicates substantial imprecision [51]. Subgroup analysis suggested a greater treatment response with tacalcitol than with calcipotriol when combined with NB-UVB (RR 2.25, 95% CI 1.60–3.17 vs. RR 1.24, 95% CI 0.85–1.81; interaction *p* = 0.02) [51]. In contrast, the addition of vitamin D analogs did not significantly improve treatment response with PUVA (RR 1.19, 95% CI 0.79–1.80) or 308-nm excimer laser (RR 1.33, 95% CI 0.75–2.33) [51]. An earlier randomized controlled study reported enhanced repigmentation and a reduction in cumulative UVA exposure with calcipotriol plus PUVA [66]; however, the pooled analysis did not demonstrate a significant overall benefit of adding vitamin D analogs to PUVA [51]. Not all studies have reported positive outcomes. A randomized right-left comparative trial by Khullar et al. found no significant difference between NB-UVB alone and NB-UVB combined with calcipotriol. However, this further illustrates the variability among individual studies, which differ in disease duration, lesion location, treatment protocols, and patient selection [67]. Overall, the available evidence suggests a potential modality-specific adjunctive benefit of topical vitamin D analogs in vitiligo, primarily when combined with NB-UVB phototherapy. In vitiligo studies, calcipotriol and tacalcitol have been administered predominantly as topical ointments, either as monotherapy or in combination with phototherapy, including NB-UVB and PUVA [51,66,67,68,69]. Interpretation of the available evidence requires caution. The meta-analysis included four parallel-group randomized controlled trials, six randomized within-patient trials, and four nonrandomized within-patient trials [51]. Statistical heterogeneity varied according to outcome: heterogeneity was low for treatment response (I^2^ = 37%) and treatment failure (I^2^ = 14%) with NB-UVB combination therapy but was substantial for excellent response (I^2^ = 64%) [51]. Risk-of-bias assessment showed that only one included study had an overall low risk of bias, whereas the remaining studies were rated as having an overall moderate risk, mainly because of high or unclear selection or attrition bias [51]. Accordingly, the GRADE assessment classified the evidence for treatment response and treatment failure with vitamin D analogs plus NB-UVB as moderate quality, while the other evaluated outcomes were supported by low- or very-low-quality evidence [51].

#### 4.3.3. Critical Appraisal

Among the non-psoriatic indications reviewed, vitiligo has one of the more extensively studied clinical evidence bases for topical vitamin D analogs. Unlike several other dermatologic disorders reviewed in this article, the biological rationale is supported by multiple randomized clinical studies and reinforced by systematic review evidence. Topical vitamin D analogs may therefore be considered potential adjunctive agents, particularly in combination with NB-UVB, with the available studies reporting predominantly minor and transient adverse events [51].

Nevertheless, important limitations remain. Most studies have evaluated calcipotriol, with comparatively little evidence available for tacalcitol and calcitriol. Treatment protocols vary considerably with respect to phototherapy schedules, treatment duration, and outcome assessment, limiting direct comparisons across studies. Furthermore, durable long-term repigmentation and relapse rates remain insufficiently characterized. Based on the currently available evidence, topical vitamin D analogs should not be considered substitutes for established vitiligo therapies. Rather, the available evidence suggests a potential modality-specific adjunctive benefit, primarily in combination with NB-UVB, while comparable benefit has not been consistently demonstrated with other phototherapy modalities.

#### 4.3.4. Future Perspectives

Future clinical research should prioritize large multicenter randomized controlled trials directly comparing individual vitamin D analogs in combination with modern vitiligo therapies, including topical Janus kinase inhibitors and emerging biologic treatments. Biomarker-guided patient selection based on VDR expression, oxidative stress profiles, or immune signatures may further optimize treatment responses. In addition, mechanistic studies investigating the interaction between vitamin D signaling, melanocyte stem cell activation, and regenerative pathways could help identify novel combination strategies capable of improving long-term repigmentation and reducing disease recurrence.

### 4.4. Basal Cell Carcinoma

#### 4.4.1. Disease Background and Biological Rationale

Basal cell carcinoma (BCC) is the most common human malignancy, accounting for approximately 36% of all non-melanoma skin cancers. Although metastatic disease is exceedingly rare, untreated BCC may cause significant local tissue destruction and functional impairment. Surgical excision remains the standard treatment for most lesions, while topical therapies, including imiquimod and 5-fluorouracil, photodynamic therapy, and Hedgehog pathway inhibitors, are reserved for selected superficial or locally advanced tumors. Nevertheless, recurrence, treatment-related morbidity, and the increasing incidence of BCC continue to stimulate interest in novel adjunctive therapeutic approaches [46,47]. The biological rationale for investigating topical vitamin D analogs in BCC extends beyond their established antiproliferative effects. Aberrant activation of the Hedgehog signaling pathway represents the principal molecular driver of BCC, and accumulating experimental evidence suggests that vitamin D signaling can modulate this pathway independently of classical VDR-mediated genomic activity. More broadly, vitamin D/VDR signaling has been implicated in the regulation of keratinocyte differentiation, apoptosis, DNA-damage responses, and inflammatory processes relevant to cutaneous carcinogenesis [46,47,70,71]. However, these broader mechanisms should be distinguished from compound-specific effects demonstrated directly in experimental BCC models. Collectively, these mechanisms provide a compelling theoretical basis for therapeutic repurposing in cutaneous oncology [46,47,70,71].

#### 4.4.2. Clinical Evidence

Clinical evidence is provided by a Phase II, single-blind randomized controlled trial of 128 patients with histologically confirmed superficial basal cell carcinoma (sBCC, *n* = 64) or nodular basal cell carcinoma (nBCC, *n*= 64) [72]. Patients were randomized into four groups that received topical diclofenac sodium 3% gel, calcitriol 3 μg/g ointment, a combination of both agents, or no treatment (control). Active treatments were applied twice daily under occlusion for 8 weeks before surgical excision. The primary outcomes were post-treatment expression of the proliferation marker Ki-67 and the anti-apoptotic marker Bcl-2, while secondary outcomes included histological tumor regression, adverse events, application-site reactions, and treatment compliance [72].

Treatment response differed according to treatment arm and BCC subtype. In sBCC, diclofenac significantly decreased Ki-67 expression (*p* < 0.001) and Bcl-2 expression (*p* = 0.001), whereas combination therapy significantly decreased Ki-67 expression (*p* = 0.012). Complete histological tumor regression was observed in 64.3% (9/14) and 43.8% (7/16) of sBCCs treated with diclofenac and combination therapy, respectively, compared with 0% in the untreated control group. In contrast, calcitriol monotherapy did not result in complete tumor regression, and post-treatment expression of Ki-67 and Bcl-2 did not differ significantly from controls. In nBCC, no significant treatment effects on Ki-67, Bcl-2, or histological tumor clearance were demonstrated [72].

Thus, while this trial provides clinical evidence for topical diclofenac in sBCC, no synergistic effect of calcitriol or its combination with diclofenac was shown [72]. Experimental studies nevertheless support specific biological activity of calcitriol in BCC models. In Ptch-mutant experimental models, calcitriol inhibited tumor-cell proliferation and Hedgehog signaling while activating VDR signaling and promoting differentiation [70,71,73]. Broader experimental evidence also indicates interactions between vitamin D/VDR signaling and Wnt/β-catenin pathways in skin tumor biology; however, these observations reflect general vitamin D/VDR biology and should not be interpreted as established effects of topical vitamin D analogs in human BCC. In the BCC trial, calcitriol 3 μg/g was administered as an ointment under occlusion [72]. Adjacent evidence from actinic keratosis has evaluated calcipotriol 0.005% combined with 5-fluorouracil 5% cream [74,75], illustrating a combination-based topical strategy in keratinocyte carcinogenesis rather than direct BCC treatment.

#### 4.4.3. Critical Appraisal

Basal cell carcinoma illustrates an important principle in translational dermatology: convincing molecular mechanisms do not necessarily translate into successful clinical therapies. Although vitamin D analogs influence several pathways involved in tumor biology, including Hedgehog signaling, apoptosis, and cellular differentiation, these effects have yet to produce consistent therapeutic benefit in patients. Current evidence does not support topical vitamin D analog monotherapy as a treatment for BCC. However, this conclusion should not be interpreted as evidence against the biological relevance of VDR signaling. Rather, it suggests that vitamin D analogs are unlikely to be sufficiently potent as single agents and may instead require combination with established topical therapies or targeted molecular inhibitors. Accordingly, vitamin D analogs should currently be regarded as investigational adjuncts in BCC rather than clinically established antitumor agents [76].

#### 4.4.4. Future Perspectives

Future research should evaluate combination strategies integrating topical vitamin D analogs with established treatments such as imiquimod, 5-fluorouracil, photodynamic therapy, or Hedgehog inhibitors. Greater understanding of the interaction between VDR signaling and oncogenic pathways, particularly Hedgehog and Wnt/β-catenin, may identify biomarkers predictive of therapeutic response. Well-designed translational studies linking molecular pathway modulation with clinical outcomes will be essential before vitamin D analogs can be considered viable adjunctive therapies in non-melanoma skin cancer.

### 4.5. Morphea (Localized Scleroderma)

#### 4.5.1. Disease Background and Biological Rationale

Morphea, or localized scleroderma, is a chronic inflammatory connective tissue disorder characterized by excessive collagen deposition, dermal fibrosis, and progressive skin sclerosis. Although its pathogenesis is incompletely understood, current evidence implicates immune dysregulation, endothelial injury, and persistent activation of fibroblasts through transforming growth factor-beta (TGF-β)-mediated signaling. Clinical management depends on disease extent and activity, ranging from topical therapies for limited superficial plaques to systemic immunosuppressive agents and phototherapy for more extensive or rapidly progressive disease [53,54]. The biological rationale for topical vitamin D analogs in morphea is particularly compelling because vitamin D signaling directly targets several pathways involved in fibrosis. Activation of the vitamin D receptor suppresses fibroblast proliferation, inhibits myofibroblast differentiation, and downregulates TGF-β/SMAD signaling, thereby reducing collagen synthesis and extracellular matrix deposition. In addition, the anti-inflammatory properties of vitamin D may limit the persistent immune activation that contributes to progressive tissue remodeling. Together, these mechanisms provide a strong scientific basis for investigating topical vitamin D analogs as antifibrotic therapies in localized scleroderma [52,53,54,55].

#### 4.5.2. Clinical Evidence

Although the available clinical reports suggest potential benefit of topical calcipotriol in morphea, the evidence remains limited and is derived primarily from small uncontrolled studies [77]. Published studies have primarily evaluated topical calcipotriol, either under occlusion or in combination with ultraviolet A1 (UVA1) phototherapy, with most reporting improvements in lesion activity and skin sclerosis. One open-label study demonstrated that twice-daily application of calcipotriol under occlusion significantly improved skin extensibility and reduced plaque induration in patients with plaque-type morphea. However, the small sample size and absence of a control group substantially limit the strength of the clinical evidence [55]. Additional evidence comes from combination therapy studies. In a prospective open-label study of 19 children with morphea, combined treatment with calcipotriol 0.005% ointment and low-dose UVA1 was associated with a 67.1% reduction in the mean clinical severity score [78]. However, because the study was uncontrolled and both interventions were administered concurrently, the independent contribution of calcipotriol and any synergistic effect with UVA1 cannot be established.

Importantly, the 2024 German S2k guideline for localized scleroderma recognizes topical vitamin D analogs as an optional therapeutic approach for limited superficial inflammatory lesions, reflecting cautious but positive expert consensus despite the limited evidence base [79]. In morphea, calcipotriol/calcipotriene 0.005% has predominantly been delivered as an ointment, either under nighttime occlusion or without occlusion in combination with UVA1 phototherapy, highlighting the potential importance of formulation and application conditions for achieving adequate cutaneous exposure [55,78].

#### 4.5.3. Critical Appraisal

Morphea represents a biologically plausible indication for topical vitamin D analog therapy. Unlike several other non-psoriatic conditions, the proposed mechanism of action directly targets a central pathogenic pathway, namely fibroblast activation mediated by TGF-β signaling, and available small uncontrolled studies have reported improvements in disease activity. However, published studies involve small patient populations, are predominantly open-label, and frequently combine vitamin D analogs with phototherapy, making it difficult to determine the independent contribution of topical treatment. Furthermore, no randomized controlled trials have directly compared vitamin D analogs with established topical therapies such as corticosteroids or tacrolimus. Consequently, current evidence supports topical vitamin D analogs as adjunctive therapies for carefully selected patients with superficial inflammatory morphea rather than as first-line treatment. Further controlled studies are required to determine their independent efficacy, optimal treatment setting, and potential benefit in combination with phototherapy.

#### 4.5.4. Future Perspectives

Future studies should prioritize multicenter randomized controlled trials comparing topical vitamin D analogs with standard topical therapies and evaluating their additive benefit when combined with UVA1 phototherapy. Incorporation of objective outcome measures, including ultrasound imaging and validated disease activity scores, would improve comparability across studies. In addition, mechanistic investigations examining the interaction between vitamin D signaling, fibroblast biology, and extracellular matrix remodeling may help identify biomarkers predictive of treatment response and facilitate more personalized therapeutic approaches.

### 4.6. Inherited Ichthyoses

#### 4.6.1. Disease Background and Biological Rationale

Inherited ichthyoses comprise a heterogeneous group of genetic disorders characterized by abnormal epidermal differentiation, defective cornification, and excessive scaling resulting from impaired skin barrier formation. Although individual subtypes differ genetically, disruption of keratinocyte maturation and terminal differentiation represents a common pathogenic feature. Current management remains largely symptomatic, relying on emollients, keratolytic agents, topical retinoids, and systemic retinoids in more severe cases. However, long-term treatment is often limited by incomplete efficacy and treatment-related adverse effects, highlighting the need for alternative therapeutic approaches [80,81]. Topical vitamin D analogs provide a biologically plausible therapeutic strategy because they directly regulate keratinocyte proliferation and terminal differentiation through activation of the vitamin D receptor. Experimental studies have demonstrated that VDR signaling increases the expression of structural proteins involved in epidermal maturation while suppressing excessive keratinocyte proliferation. These mechanisms closely target the fundamental pathological abnormalities underlying inherited ichthyoses and therefore represent one of the most direct examples of disease-specific therapeutic repurposing beyond psoriasis [80,81].

#### 4.6.2. Clinical Evidence

Clinical evaluation of topical vitamin D analogs in inherited ichthyoses has primarily focused on calcipotriol. Small randomized and vehicle-controlled studies have reported improvements in scaling, epidermal thickness, and skin appearance following topical treatment; however, the magnitude and consistency of response have varied among patients and ichthyosis subtypes [82,83]. In one placebo-controlled clinical trial, calcipotriol reduced scaling severity compared with vehicle treatment and was associated with changes in histological markers of epidermal differentiation [82,83]. These findings provide preliminary clinical support for the ability of topical calcipotriol to modulate abnormal epidermal differentiation; however, they should be interpreted cautiously given the small study populations, heterogeneity of the included ichthyosis subtypes, and limited duration of follow-up. Treatment responses have varied across different forms of inherited ichthyosis, consistent with the substantial genetic and phenotypic heterogeneity of these disorders.

Consequently, the available findings cannot be generalized across all inherited ichthyosis subtypes. Clinical studies have primarily employed calcipotriol 0.005% (50 μg/g) ointment [82,83].

#### 4.6.3. Critical Appraisal

Inherited ichthyoses provide a biologically plausible setting for topical vitamin D analog therapy because regulation of keratinocyte differentiation directly relates to an important component of disease pathophysiology. However, the clinical evidence remains limited. Available studies involve small patient populations, include heterogeneous ichthyosis subtypes, and report variable treatment responses, making it difficult to determine which patient groups are most likely to benefit. Nevertheless, clinical application is constrained by an important safety consideration. The potential for systemic calcium disturbances is particularly relevant when large body-surface areas are treated. Although clinically significant hypercalcemia appears uncommon, a hypercalcemic crisis has been reported in a 17-year-old patient following excessive topical calcipotriol use for ichthyosis [45].

Consequently, careful monitoring of cumulative dose, treatment duration, and serum calcium concentrations is recommended when large treatment areas are involved [80]. Accordingly, topical vitamin D analogs should currently be considered adjunctive therapies for carefully selected patients with localized or moderately extensive disease rather than routine treatment for generalized ichthyosis.

#### 4.6.4. Future Perspectives

Future clinical studies should evaluate whether lower-concentration formulations, intermittent dosing schedules, or combination regimens with topical retinoids and barrier-repair therapies can maintain therapeutic efficacy while reducing systemic calcium exposure. Given the marked genetic heterogeneity among inherited ichthyoses, genotype-specific treatment strategies and biomarker-guided patient selection may further optimize clinical outcomes. Longer-term studies evaluating both efficacy and systemic safety will be essential before broader clinical recommendations can be established.

### 4.7. Alopecia Areata

#### 4.7.1. Disease Background and Biological Rationale

Alopecia areata (AA) is an autoimmune, non-scarring hair loss disorder characterized by T-cell-mediated destruction of anagen hair follicles and collapse of the immune privilege normally maintained within the follicular microenvironment. Current therapies include topical and intralesional corticosteroids, topical immunotherapy, and Janus kinase (JAK) inhibitors, although treatment responses remain variable. The biological rationale for topical vitamin D analogs is supported by evidence that hair follicles express functional vitamin D receptors (VDR), which are essential for normal hair cycling and follicular differentiation. In addition, vitamin D signaling modulates immune responses by promoting regulatory T-cell activity and suppressing inflammatory cytokines implicated in AA pathogenesis [3,24,25,84,85]. These observations provide a mechanistic basis for investigating topical vitamin D analogs as adjunctive therapies in alopecia areata [84,85,86,87,88].

#### 4.7.2. Clinical Evidence

Clinical evidence supporting topical vitamin D analogs in AA remains limited and heterogeneous. Published studies consist primarily of small prospective trials, observational studies, and case reports evaluating topical calcipotriol, either alone or in combination with established therapies. Several small clinical studies have reported partial hair regrowth following topical calcipotriol treatment, particularly in patients with localized patch-type alopecia areata. In some comparative studies, combination therapy with topical corticosteroids appeared to produce greater clinical improvement than either treatment alone, suggesting a potential synergistic effect between immunosuppressive therapy and VDR-mediated immune modulation [84,85,86,87,88]. However, clinical responses have been inconsistent, and the available studies are limited by small sample sizes, short treatment duration, and heterogeneous outcome measures. To date, no adequately powered multicenter randomized controlled trial has established the efficacy of topical vitamin D analogs in alopecia areata. Topical calcipotriol 0.005% has been investigated in several conventional formulations in alopecia areata, including ointment, cream, and lotion, reflecting the need for vehicles with appropriate spreadability and follicular delivery characteristics in hair-bearing areas [84,85,86,87,88].

#### 4.7.3. Critical Appraisal

The available evidence suggests that topical vitamin D analogs possess an interesting biological rationale but only modest clinical support for use in alopecia areata. Unlike vitiligo, where mechanistic and clinical findings are largely concordant, evidence in AA remains preliminary and insufficient to define a clear therapeutic role. An additional limitation is the uncertainty regarding the mechanism responsible for the reported clinical improvements. It remains unclear whether treatment responses primarily reflect direct modulation of hair follicle biology, restoration of immune privilege, suppression of autoimmune inflammation, or a combination of these effects. Furthermore, most available studies have evaluated calcipotriol, leaving the potential role of tacalcitol and calcitriol almost entirely unexplored. At present, topical vitamin D analogs should therefore be regarded as experimental adjunctive therapies rather than evidence-based treatment options for alopecia areata.

#### 4.7.4. Future Perspectives

Future investigations should focus on well-designed randomized controlled trials comparing vitamin D analogs with current standard therapies and evaluating their potential role in combination with topical corticosteroids or JAK inhibitors. Greater understanding of VDR signaling within the hair follicle stem cell niche, together with biomarker-guided patient stratification based on vitamin D receptor expression or immune signatures, may help identify patients most likely to benefit from VDR-targeted therapy. Such studies will be essential to determine whether the promising mechanistic rationale can be translated into clinically meaningful improvements in hair regrowth and disease remission.

### 4.8. Emerging Clinical Applications

Although most research has focused on acne, vitiligo, morphea, and inherited ichthyoses, topical vitamin D analogs have also been investigated in other dermatologic conditions, including cutaneous lichen planus [89]. Additional potential applications in wound healing, tissue repair, and scar modulation have been proposed on mechanistic or preliminary experimental grounds; however, clinical evidence remains insufficient to establish their therapeutic role. Across these conditions, the proposed therapeutic effects are largely attributed to the pleiotropic actions of vitamin D signaling, including modulation of cutaneous inflammation, enhancement of epidermal differentiation, regulation of immune responses, and promotion of tissue repair. Although these biological mechanisms provide a reasonable scientific rationale, current evidence remains insufficient to support routine clinical use outside carefully selected cases or research settings [1,2,3]. Future investigations should prioritize adequately powered randomized controlled trials, standardized clinical outcome measures, and biomarker-based patient selection to determine whether these preliminary observations can be translated into clinically meaningful therapeutic applications. Until such evidence becomes available, the use of topical vitamin D analogs in these emerging indications should be regarded as investigational.

## 5. Comparative Analysis of Clinical Evidence

The available literature demonstrates substantial heterogeneity in both the biological rationale and the quality of clinical evidence supporting the use of topical vitamin D analogs beyond psoriasis. Although activation of the vitamin D receptor influences multiple pathogenic pathways, including epidermal differentiation, immune regulation, oxidative stress, fibrosis, and tumor biology, translation of these molecular mechanisms into consistent clinical benefit varies considerably among different dermatologic disorders. Consequently, therapeutic recommendations should be guided not only by mechanistic plausibility but also by the strength and consistency of available clinical evidence [1,2,3,4]. Among all indications reviewed, vitiligo currently possesses the most robust evidence base. Randomized clinical studies and systematic review evidence suggest a potential adjunctive benefit of topical vitamin D analogs, particularly when combined with NB-UVB phototherapy [51,66,67,68,69]. However, the magnitude and consistency of benefit vary among studies and according to phototherapy modality. These findings suggest that vitamin D analogs function most effectively as adjunctive rather than stand-alone therapies, potentiating the benefits of phototherapy through complementary effects on melanocyte survival, oxidative stress, and immune regulation [21,22,24,25,51]. Morphea also demonstrates a favorable balance between mechanistic rationale and clinical outcomes. Although evidence is limited to small prospective studies, improvements in skin induration, extensibility, and inflammatory activity have been reported in small uncontrolled studies, including studies evaluating topical calcipotriol alone or in combination with UVA1 phototherapy. The inclusion of topical vitamin D analogs as an optional therapeutic approach in the recent German S2k guideline further supports their potential role in carefully selected patients with localized superficial inflammatory disease [53,54,55,78,79]. For acne vulgaris and inherited ichthyoses, the available evidence is encouraging but remains moderate. In acne, topical vitamin D analogs demonstrate measurable anti-inflammatory activity and improvements in lesion counts; however, existing studies consistently show inferior efficacy compared with topical retinoids, supporting their role primarily as adjunctive therapies rather than first-line treatment [30,31,32,57,58]. In inherited ichthyoses, the close relationship between VDR signaling and keratinocyte differentiation provides one of the strongest mechanistic rationales among all reviewed conditions. Nevertheless, concerns regarding systemic calcium absorption during treatment of extensive body surface areas continue to limit widespread clinical application despite preliminary evidence of clinical benefit [80,81,82,83]. Conversely, atopic dermatitis, alopecia areata, and basal cell carcinoma illustrate the challenges of translating promising experimental findings into clinical practice. In atopic dermatitis, the paradoxical induction of TSLP-mediated inflammation by topical vitamin D analogs in experimental models raises questions regarding their therapeutic role [6]. Clinical evidence in alopecia areata remains limited to relatively small studies with heterogeneous designs and outcomes [84,85,86,87,88]. Similarly, in basal cell carcinoma, clinical investigation has failed to reproduce fully the encouraging antitumor activity observed in preclinical models [70,71,72]. Clinical outcome heterogeneity should primarily be interpreted within individual disease categories rather than through direct comparison across different dermatological conditions. The included studies necessarily used disease-specific endpoints, such as lesion counts in acne, repigmentation outcomes in vitiligo, measures of skin induration and disease activity in morphea, scaling severity in inherited ichthyoses, and hair-regrowth or SALT-based outcomes in alopecia areata. These endpoints assess distinct clinical domains and are therefore not directly comparable across diseases. Within each condition, heterogeneity may additionally arise from differences in disease subtype and severity, anatomical site, treatment protocol and duration, comparator, and outcome definition. Accordingly, cross-disease comparisons in this review are intended to describe the nature and strength of the available evidence rather than to compare the magnitude of therapeutic effects between conditions. The principal clinical studies evaluating topical vitamin D analogs across the non-psoriatic dermatologic conditions reviewed, together with their main outcomes and evidence certainty, are summarized in Table 3.

An additional observation emerging from this review is the overwhelming predominance of calcipotriol in the published literature. Comparatively few studies have evaluated tacalcitol or calcitriol, making it difficult to determine whether observed differences in clinical outcomes reflect genuine pharmacological variation or simply disparities in research activity. Likewise, no adequately powered head-to-head comparative trials have evaluated the three available analogs across non-psoriatic indications. Consequently, current analog selection continues to rely largely on pharmacological characteristics, anatomical treatment site, local tolerability, and clinician experience rather than comparative clinical evidence [4,5,17,18,19,20]. Overall, the available evidence supports topical vitamin D analogs primarily as adjunctive therapies capable of complementing established treatment strategies rather than replacing standard-of-care interventions. Their greatest therapeutic potential appears to lie in combination regimens that exploit their pleiotropic biological effects while maintaining the favorable safety profile established during decades of psoriasis management. Future clinical development should therefore prioritize disease-specific randomized controlled trials, direct comparisons among available vitamin D analogs, standardized outcome measures, and prospective evaluation of potential biomarkers of treatment response to support future personalized therapeutic approaches [50,57,63,83].

## 6. Current Challenges and Future Perspectives

Despite the expanding interest in topical vitamin D analogs beyond psoriasis, several important challenges continue to limit their broader clinical application. Foremost among these is the limited availability of high-quality clinical evidence. Most published studies involve small patient cohorts, single-center designs, short treatment durations, and heterogeneous outcome measures, making direct comparison across studies difficult and limiting the strength of current therapeutic recommendations [51,55,57,58,63,78,79,82,83,84,85,86,87,88]. Another major limitation is the lack of direct comparative studies evaluating the three currently available topical vitamin D analogs. Although calcipotriol, tacalcitol, and calcitriol differ in receptor affinity, metabolic stability, local tolerability, and calcemic potential, virtually all non-psoriatic clinical studies have investigated calcipotriol, while evidence for tacalcitol and calcitriol remains comparatively scarce. Consequently, current analog selection is based largely on extrapolation from psoriasis, regional availability, and clinician preference rather than disease-specific comparative evidence [4,5,17,18,20]. Clinical outcome heterogeneity should primarily be interpreted within individual disease categories rather than through direct comparison across different dermatological conditions. The included studies necessarily used disease-specific endpoints, such as lesion counts in acne, repigmentation outcomes in vitiligo, measures of skin induration and disease activity in morphea, scaling severity in inherited ichthyoses, and hair-regrowth or SALT-based outcomes in alopecia areata. These endpoints assess distinct clinical domains and are therefore not directly comparable across diseases. Within each condition, heterogeneity may additionally arise from differences in disease subtype and severity, anatomical site, treatment protocol and duration, comparator, and outcome definition. Accordingly, cross-disease comparisons in this review are intended to describe the nature and strength of the available evidence rather than to compare the magnitude of therapeutic effects between conditions. Future research should focus on adequately powered multicenter randomized controlled trials, direct comparisons among vitamin D analogs, and optimization of combination treatment strategies. Integration of topical vitamin D analogs with phototherapy, topical immunomodulators, retinoids, and emerging targeted therapies may further enhance therapeutic efficacy while minimizing adverse effects. Biomarker-guided treatment represents a potentially valuable future research direction. Candidate biomarkers, including VDR expression patterns, cytokine profiles, genetic polymorphisms, and disease-specific molecular signatures, warrant prospective evaluation as potential markers of treatment response [90,91,92,93]. However, these biomarkers have not yet been adequately validated for predicting clinical response to topical vitamin D analogs. Prospective studies are required to determine their predictive value and potential utility for patient stratification and personalized treatment strategies.

**Figure 4:**
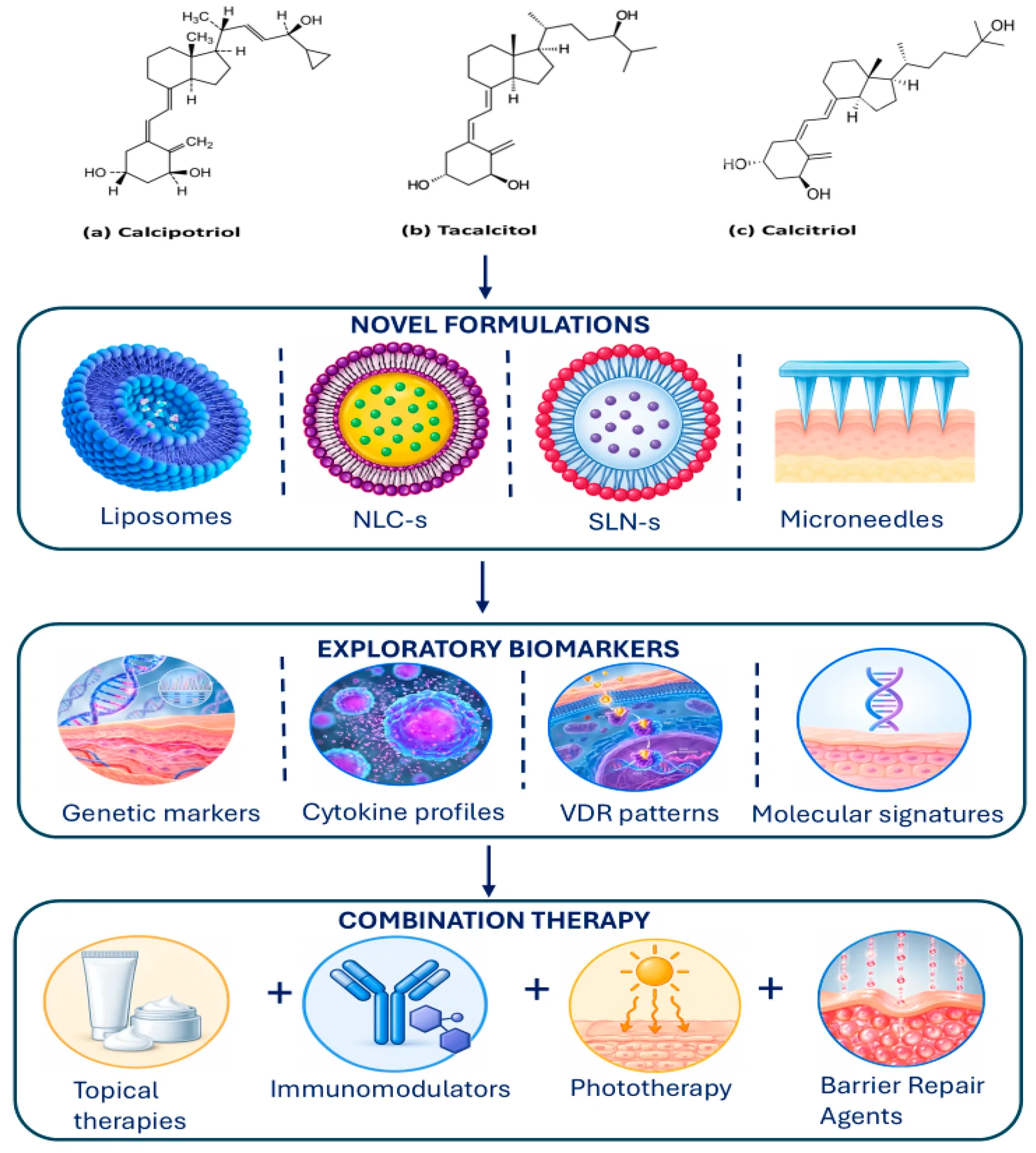
Future directions for the clinical development of topical vitamin D analogs beyond psoriasis.

Finally, advances in formulation science, including nanocarrier systems, lipid-based delivery platforms, and controlled-release topical formulations, may improve cutaneous penetration, increase local drug retention, and reduce systemic exposure. Such innovations could expand the therapeutic utility of vitamin D analogs, particularly in diseases requiring prolonged treatment or involving extensive body surface areas. Continued collaboration between basic scientists, formulation researchers, and clinical dermatologists will therefore be essential to translate the promising biological properties of topical vitamin D analogs into evidence-based therapeutic strategies. Collectively, these advances indicate that the future development of topical vitamin D analogs will depend on the integration of innovative pharmaceutical formulations, prospective validation of candidate biomarkers, and rational combination therapies. The principal directions for future research are summarized in Figure 4.

## 7. Conclusions

Topical vitamin D analogs have emerged as promising candidates for therapeutic repurposing beyond their established role in psoriasis. Advances in the understanding of vitamin D receptor (VDR) biology have demonstrated that these agents regulate multiple cellular processes, including keratinocyte differentiation, immune homeostasis, antimicrobial defense, melanocyte survival, extracellular matrix remodeling, and tumor-associated signaling. These pleiotropic biological effects provide a compelling mechanistic rationale for investigating topical vitamin D analogs across a broad spectrum of inflammatory, pigmentary, fibrotic, and neoplastic skin disorders. However, the translation of these molecular mechanisms into clinical practice remains highly disease-dependent. Among the conditions reviewed, vitiligo currently has the strongest clinical evidence, particularly when topical vitamin D analogs are combined with narrowband ultraviolet B phototherapy. Morphea also shows encouraging clinical outcomes supported by a strong anti-fibrotic rationale, although evidence remains limited to relatively small studies. In contrast, acne vulgaris and inherited ichthyoses show moderate but promising evidence supporting adjunctive use, whereas atopic dermatitis, alopecia areata, and basal cell carcinoma remain characterized by a substantial gap between experimental findings and established clinical efficacy. One of the most important observations emerging from this review is the marked imbalance in the available literature. Most studies have evaluated calcipotriol, whereas direct evidence regarding tacalcitol and calcitriol remains scarce. Moreover, adequately powered comparative studies have not established whether pharmacological differences among currently available vitamin D compounds translate into clinically meaningful differences in efficacy or safety. Consequently, treatment selection for non-psoriatic indications continues to rely largely on extrapolation from psoriasis, pharmacological principles, and limited disease-specific evidence. Future progress will require adequately powered multicenter randomized controlled trials, standardized outcome measures, direct comparisons among available vitamin D compounds, and prospective validation of candidate biomarkers of treatment response. Importantly, therapeutic development should not focus exclusively on identifying new biological indications. Optimization of cutaneous drug delivery may be equally important, as vehicle composition, drug release, skin retention, penetration depth, and targeted deposition within disease-relevant compartments can determine whether pharmacological activity translates into clinical efficacy. The optimal delivery target may differ substantially among diseases, ranging from the pilosebaceous unit in acne and the hair follicle in alopecia areata to the viable epidermis in vitiligo and deeper dermal compartments in morphea. Advanced formulation strategies, including nanocarrier-based and other targeted cutaneous delivery systems, therefore represent an important area for pharmaceutical development, although their clinical value for vitamin D analogs remains to be established. Overall, topical vitamin D analogs should currently be regarded primarily as adjunctive rather than replacement therapies for most non-psoriatic skin diseases. Their established dermatologic use, pleiotropic biological activity, and compatibility with existing therapeutic modalities nevertheless support continued development. Integration of pharmacological understanding with formulation science, cutaneous pharmacokinetics, disease-specific targeting, and rigorous clinical evaluation will be essential to determine whether these agents can evolve into evidence-based components of personalized dermatologic therapy.

## Figures and Tables

**Table 1:** Pharmacological and pharmaceutical characteristics of topical vitamin D compounds.

Property	Calcipotriol	Tacalcitol	Calcitriol	References
VDR activity	High	High	Native VDR ligand	[4,5,17]
Calcemic potential	Low	Low	Higher relative potential	[4,5,17,19]
Common topical concentration	50 µg/g (0.005%)	4 µg/g	3 µg/g	[4,17,19]
Application frequency	Once or twice daily	Once daily	Twice daily	[4,17,19]
Systemic calcium considerations	Low at recommended topical exposure	Low at recommended topical exposure	Greater caution with extensive/prolonged application	[4,17,19,21]
Local tolerability (face/flexures)	Irritation may occur, particularly at sensitive sites	Generally, well tolerated	Generally favorable at sensitive/flexural sites	[4,17,19]
Principal established dermatologic indication	Plaque psoriasis	Plaque psoriasis	Plaque psoriasis	[4,17,19]
Common topical dosage forms	Cream, ointment, solution, and other topical vehicles	Ointment	Ointment	[4,17,19,21]

Note: The concentrations, application frequencies, and pharmaceutical formulations presented in this table reflect commonly used and/or authorized topical preparations reported in the cited sources. Regulatory approval, available formulations, and recommended dosing may vary according to compound, product, and jurisdiction. Current UK-authorized product information is discussed separately where relevant [10,11,12].

**Table 2:** Advanced cutaneous delivery systems investigated for topical vitamin D compounds.

Delivery System	Vitamin D Compound(s)	Experimental Model	Particle Size	EE/ Loading	Skin Delivery Findings	Main Therapeutic Findings	Development Stage
Liposomal phospholipid preparations [36]	Calcitriol; tacalcitol	Mouse-tail test	NR	NR	Liposomal incorporation enhanced local pharmacological activity compared with petrolatum/non-liposomal phospholipid vehicles	Greater antiparakeratotic activity than non-liposomal preparations	Preclinical, in vivo
PEGylated liposomes (PEG-DSPE) [37]	Calcipotriol	Excised porcine skin	93.1–95.4 nm *	NR	1 mol% PEG-DSPE significantly increased deposition in the stratum corneum; small unilamellar vesicles enhanced penetration compared with large multilamellar vesicles	Pharmacological efficacy not evaluated	Preclinical, ex vivo
NLCs (Precirol ATO 5/squalene) [38]	Calcipotriol + methotrexate	In vitro skin permeation; hyperproliferative skin; in vivo CLSM	270–320 nm	NR	Calcipotriol flux 0.62–1.08 µg/cm^2^/h vs. 0.72 µg/cm^2^/h with control; co-loading reduced calcipotriol permeation	Successful simultaneous topical delivery	Preclinical
SLNs incorporated into Carbopol gel [39]	Calcipotriol + betamethasone dipropionate	Skin permeation/distribution; HaCaT cells; mouse-tail model	NR	NR	Drugs were predominantly localized in epidermal/dermal regions, with negligible receptor-compartment amounts	Experimental antiproliferative and antiparakeratotic activity	Preclinical
Nanoemulsion in Carbopol 980 gel [40]	Calcipotriol + clobetasol propionate	HaCaT cells; skin penetration; IMQ-induced psoriasis in mice	NR	NR	Increased uptake and penetration into stratum corneum and viable skin compared with free drugs	Greater experimental antipsoriatic activity than free drugs/comparator	Preclinical
SLNs/NLCs in Carbopol 934P-NF gel [41]	Calcipotriol + cyclosporine	Ex vivo dermatokinetics; HaCaT cells; IMQ-induced psoriasis in mice	<100 nm	High; exact values formulation-dependent	Deeper and more localized epidermal penetration than free drugs	NLC gel showed greater experimental activity than SLN formulation	Preclinical
Nanosuspension-loaded PVP/PVA dissolving microneedles [43]	Calcipotriol	In vitro delivery; IMQ-induced psoriasis in rats	** 211 ± 2 nm	NR	Microneedles enabled direct local delivery across the stratum corneum	Activity comparable with marketed Daivonex ointment	Preclinical

Abbreviations: CLSM, confocal laser scanning microscopy; EE, encapsulation efficiency; IMQ, imiquimod; NLC, nanostructured lipid carrier; PEG-DSPE, poly (ethylene glycol)-distearoylphosphoethanolamine; PVA, poly (vinyl alcohol); PVP, poly(vinylpyrrolidone); SLN, solid lipid nanoparticle. * Particle size refers to the extruded PEGylated liposomal formulations; larger non-extruded vesicles were >500 nm. ** Particle size refers to the calcipotriol nanosuspension incorporated into the microneedle system. Despite these promising findings, the clinical advantages of advanced delivery systems over conventional vitamin D formulations remain to be established. Continued optimization of topical formulations, together with advances in nanotechnology and biomaterial-based delivery systems, may substantially expand the therapeutic potential of vitamin D analogs beyond their current indications and improve their integration into personalized dermatologic therapy [6,7,8,9].

**Table 3:** Summary of clinical evidence for topical vitamin D analogs in non-psoriatic dermatologic conditions.

Condition	Analog	Design	N	Formulation/Protocol	Comparator	Main Outcome	Key Result	Evidence Certainty	Ref
Acne vulgaris	Calcipotriol	Triple-blind, randomized, two-group, split-face trial	60 (30 per group)	Group 1: calcipotriol 0.005% cream vs. placebo on contralateral facial sides; Group 2: adapalene 0.1% gel vs. calcipotriol 0.005% cream on contralateral facial sides; 8 wk	Adapalene 0.1% gel vs. placebo (parallel split-face groups)	Inflammatory & non-inflammatory lesion count; Physician & Patient Global Assessment	Significant lesion reduction with calcipotriol; adapalene superior (*p* = 0.001); highest patient satisfaction with adapalene	Moderate	[57]
Acne vulgaris	Calcipotriol	Prospective split-face study	40	0.005% cream × 2 months; once daily application	Adapalene 0.1% gel (contralateral side)	Inflammatory & non-inflammatory lesion count; histology	Significant reduction in both inflammatory and non-inflammatory lesions on calcipotriol side (*p* = 0.0001)	Low	[58]
Chronic hand eczema * (adjacent evidence)	Calcipotriol	Comparative clinical study	NR	Calcipotriol ointment applied to affected hands for study duration	Desoximetasone (topical corticosteroid)	Clinical severity score (hand eczema)	Calcipotriol showed efficacy comparable to desoximetasone in chronic hand eczema	Low	[63]
Vitiligo	Calcipotriol/Tacalcitol	Systematic review & meta-analysis (14 RCTs/within patient studies)	642	Topical vitamin D analog (calcipotriol or tacalcitol) + NB-UVB or PUVA or excimer laser	Phototherapy alone	Treatment response (≥50% repigmentation); relative risk	NB-UVB combination superior (RR 1.67, 95% CI 1.21–2.31); tacalcitol > calcipotriol (RR 2.25 vs. 1.24, *p* = 0.02); no benefit added to PUVA or excimer	Moderate	[51]
Vitiligo	Calcipotriol	Placebo-controlled double-blind left–right RCT	27	Topical calcipotriol + oral PUVA vs. placebo + oral PUVA	Oral PUVA + placebo cream	% repigmentation (initial & complete); cumulative UVA dose; number of sessions	81% initial vs. 7% placebo; 63% complete vs. 15% placebo; lower cumulative UVA dose (232.8 vs. 259.9 J/cm^2^); fewer sessions required	Moderate	[66]
Vitiligo	Tacalcitol	Randomized split-body trial	32	Tacalcitol OD + NB-UVB twice weekly	NB-UVB alone (contralateral side)	Repigmentation rate and response	Significant improvement in repigmentation and response rates with tacalcitol + NB-UVB vs. NB-UVB alone	Moderate	[68]
Vitiligo	Calcipotriol	Right–left comparative RCT (24 wk)	27	Calcipotriol 0.005% ointment BID + NB-UVB × 24 wk	NB-UVB alone (contralateral side)	% repigmentation (Leukoderma & Body Score); IGA	No significant added benefit: mean L&B-score reduction 49% vs. 51.4% NB-UVB alone (*p* = 0.557); IGA 2.6 vs. 2.7 (*p* = 0.821)	Moderate	[67]
Vitiligo	Calcipotriol/Betamethasone	RCT	41	Calcipotriol/betamethasone ointment + NB-UVB × 6 months	Tacrolimus 0.1% ointment + NB-UVB	% repigmentation for 6 months	54.7% repigmentation (calcipotriol/betamethasone + NB-UVB) vs. 45.6% (tacrolimus + NB-UVB); difference not statistically significant	Moderate	[58]
Basal cell carcinoma	Calcitriol	Phase II RCT	128	Calcitriol 3 µg/g ointment BID under occlusion × 8 wk; arms: calcitriol alone, diclofenac 3% alone, combination, or no topical treatment	Diclofenac 3%; combination; no-treatment control	Histologic regression rate; Ki-67 & Bcl-2 expression	Calcitriol monotherapy: ineffective for sBCC and nBCC; diclofenac 3%: 64.3% histologic regression in sBCC; combination: 43.8% regression in sBCC; no benefit for nBCC in any arm	Moderate	[72]
Actinic keratosis/keratinocyte cancer prevention (adjacent evidence)	Calcipotriol	Randomized field therapy trial	131	Calcipotriol 0.005% + 5-fluorouracil 5% cream × 4 days	Vehicle + 5-FU cream	AK clearance; local immune response/CD4+ T-cell infiltration	Marked reduction in AK burden and induction of a robust T-cell response with calcipotriol + 5-FU compared with 5-FU control.	Moderate	[74]
Ichthyosis (lamellar & BIE)	Calcipotriol	Double-blind bilaterally paired study	6	Calcipotriol 0.005% ointment vs. vehicle, applied to contralateral body halves	Vehicle (contralateral side)	Scaling, clinical improvement; serum calcium	Unilateral improvement on calcipotriol side in 3/4 lamellar ichthyosis patients and 1 BIE patient; no adverse laboratory findings	Low	[82]
Ichthyosis (multiple subtypes)	Calcipotriol	RCT double-blind vehicle-controlled right/left (12 wk)	67 (27 ichthyosis, 20 PPK, 9 KP, 11 other)	Calcipotriol ointment 50 µg/g up to 120 g/wk vs. vehicle	Vehicle (contralateral side)	Scaling severity across keratinization subtypes	Statistically significant reduction in scaling in ichthyosis variants; no benefit in palmoplantar keratoderma or keratosis pilaris	Moderate	[83]
Morphea/Linear scleroderma	Calcipotriol	Open-label pilot study	12 (aged 12–38 yrs)	Calcipotriene 0.005% ointment BID under nighttime occlusion × 3 months; prior TCS failure	Within-subject pre/post (no control arm)	Skin extensibility; erythema; induration; dyspigmentation; serum calcium; PTH	All 12 patients improved significantly in skin extensibility; notable reductions in erythema, induration, dyspigmentation; no perturbation in serum calcium or PTH	Very low	[55]
Morphea (childhood)	Calcipotriol	Open-label + low-dose UVA1 (12 wk)	19 (pediatric)	Calcipotriol 0.005% ointment BID (unoccluded) + UVA1 phototherapy 20 J/cm^2^ 4 times/weekly × 12 wk	No control arm	Clinical severity score (relative reduction)	Mean clinical severity score decreased from 7.3 ± 0.9 at baseline to 2.4 ± 0.9 after combined treatment (67.1% relative reduction); uncontrolled design precludes attribution of the improvement to calcipotriol or demonstration of synergy with UVA1	Very low	[78]
Alopecia areata	Calcipotriol	RCT (3 months)	N = 60	Topical calcipotriol; NB-UVB; calcipotriol + NB-UVB	Placebo; active-treatment comparisons	SALT score; serum vitamin D_3_ levels measured at baseline and after 3 months	Both treatments significantly improved SALT scores; associated rise in serum vitamin D3; no significant difference between groups	Moderate	[85]
Alopecia areata	Calcipotriol	Intrasubject pilot study (12 wk)	35	Calcipotriol 0.005% ointment BID × 12 wk	Clobetasol propionate 0.05% ointment BID × 12 wk on contralateral sites	≥75% hair regrowth	≥75% regrowth at 62.9% of calcipotriol-treated sites vs. 45.7% of clobetasol-treated sites; numerical advantage, not statistically significant	Low	[86]
Alopecia areata	Calcipotriol	Prospective pilot study (3 months)	22	Calcipotriol lotion BID × 3 months	None	Hair regrowth; SALT score; baseline serum vitamin D level	Terminal hair regrowth in 13/22 participants (59.1%); more robust response in patients with lower baseline serum vitamin D	Very low	[87]
Alopecia areata	Calcipotriol	Retrospective study (12 wk)	48	Calcipotriol cream BID × 12 wk	None	SALT score; hair-regrowth rate	Overall response rate 69.2%; ≥75% regrowth in 62.5%; complete hair restoration in 27.1%	Very low	[88]
Cutaneous lichen planus	Calcipotriol	Randomized open-label trial (12 wk)	31	Calcipotriol ointment 50 µg/g BID × 12 wk	Betamethasone valerate 0.1% ointment BID × 12 wk	Lesion flattening; pigmentary clearance; pruritus (VAS)	Calcipotriol not superior to betamethasone valerate for lesion flattening, pigmentation, or pruritus relief; comparable efficacy	Low	[89]

* Chronic hand eczema is presented as adjacent evidence because of shared inflammatory and barrier-related features with atopic dermatitis but does not constitute direct clinical evidence for AD. Evidence certainty was qualitatively classified as high, moderate, low, or very low according to study design, sample size, presence of a comparator, consistency of findings, and major methodological limitations; this classification does not represent a formal GRADE assessment. Effect estimates and 95% confidence intervals are reported where available from the original studies or systematic reviews. Additional population characteristics, adverse events, follow-up, and study-specific limitations are described in the corresponding disease-specific sections of the text.

## Data Availability

No new data were created or analyzed in this study. Data sharing is not applicable to this article.

## Abbreviations

The following abbreviations are used in this manuscript:

AK	Actinic keratosis
BID	Twice daily
BIE	Bullous ichthyosiform erythroderma
Wk	Week
BCC	Basal cell carcinoma
IGA	Investigator Global Assessment
NB-UVB	Narrowband ultraviolet B
NR	Not reported
OD	Once daily
PPK	Palmoplantar keratoderma
PTH	Parathyroid hormone
PUVA	Psoralen plus ultraviolet A
RCT	Randomized controlled trial
SALT	Severity of Alopecia Tool
sBCC	Superficial basal cell carcinoma
nBCC	Nodular basal cell carcinoma
TCS	Topical corticosteroid
UVA1	Ultraviolet A1
VAS	Visual analog scale
5-FU	5-fluorouracil
